# A Curve Shaped Description of Large Networks, with an Application to the Evaluation of Network Models

**DOI:** 10.1371/journal.pone.0019784

**Published:** 2011-05-17

**Authors:** Xianchuang Su, Xiaogang Jin, Yong Min, Linjian Mo, Jiangang Yang

**Affiliations:** 1 Institute of Artificial Intelligence, College of Computer Science, Zhejiang University, Hangzhou, Zhejiang, China; 2 Ningbo Institute of Technology, Zhejiang University, Ningbo, Zhejiang, China; Dana-Farber Cancer Institute, United States of America

## Abstract

**Background:**

Understanding the structure of complex networks is a continuing challenge, which calls for novel approaches and models to capture their structure and reveal the mechanisms that shape the networks. Although various topological measures, such as degree distributions or clustering coefficients, have been proposed to characterize network structure from many different angles, a comprehensive and intuitive representation of large networks that allows quantitative analysis is still difficult to achieve.

**Methodology/Principal Findings:**

Here we propose a mesoscopic description of large networks which associates networks of different structures with a set of particular curves, using breadth-first search. After deriving the expressions of the curves of the random graphs and a small-world-like network, we found that the curves possess a number of network properties together, including the size of the giant component and the local clustering. Besides, the curve can also be used to evaluate the fit of network models to real-world networks. We describe a simple evaluation method based on the curve and apply it to the *Drosophila melanogaster* protein interaction network. The evaluation method effectively identifies which model better reproduces the topology of the real network among the given models and help infer the underlying growth mechanisms of the *Drosophila* network.

**Conclusions/Significance:**

This curve-shaped description of large networks offers a wealth of possibilities to develop new approaches and applications including network characterization, comparison, classification, modeling and model evaluation, differing from using a large bag of topological measures.

## Introduction

Networks have been widely used as a concise mathematical representation of the structure of systems with interacting objects [1]–[4]. Protein-protein interaction networks, brain networks, scientific collaboration networks, the Internet and the World Wide Web are a few examples.

Decades ago, the study of graph theory focused on the analysis of small networks, or regular graphs such as a lattice. One could easily lay out the network on a piece of paper and visually investigate its features. However, real-world networks studied in recent years often involve thousands or millions of vertices and edges. Networks on this scale cannot be easily represented in a way that allows quantitative analysis to be conducted by eye [5]. Instead of network drawing, the current understanding of network structure relies mainly on specific properties, measures or statistics, such as degree distributions [6], [7], community structure measurements [8]–[10], or motif counts [11]. But one may note that specific properties characterize the structure of networks point-by-point. We are used to carrying a large bag of measures to describe a network. A good description or representation of network which holds more complete topological information in one bag may provide a clear intuitive understanding of network and reflect some special structural features, such as the curved landscape of the World Wide Web [12], cartographic representation of complex networks [13] and circular perspective drawings of protein interaction networks [14].

With this view in mind, we propose a mesoscopic description of large networks by using breadth-first search. It serves as a bridge linking networks of different structures with a set of particular curves. We use curves of this kind to represent the corresponding networks and refer to them as the *characteristic curves*. Then we apply this curve shaped description to both random graphs and lattice embedded random regular graphs, and derive the expressions of their curves. The curve expression possesses a number of network properties in one bag, such as the size of the giant component and the local clustering. Interestingly, it shows that not only homogeneous random graphs appear to have a power-law degree distribution 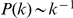 under traceroute sampling [15], [16], but a small-world-like network also does.

Moreover, characteristic curves or functions shaped by network structures can be used to compare networks comprehensively, e.g., the mesoscopic response function [17] resembling fingerprints. The network structural comparison has many applications. A useful one is to evaluate how well a network model fits a real-world network by comparing the network generated by the model with that of the real world. In recent years, network modeling has been attracting tremendous attention. Various models have been proposed to reproduce the topology of the real-world networks to infer their underlying growth mechanisms. Among the notable ones are the preferential attachment model [18], [19] and the small-world model [20]. Even a specific real-world network often has a variety of well-fitting models. Take protein-protein interaction (PPI) networks as an example, there are multiple models of widely varying mechanisms (e.g. [21]–[25],) that perfectly fit the real PPI data in terms of selected network properties, such as the degree distributions or the clustering coefficients. However, questions arise: among so many good models, which one best reproduces the structure of the real data? Which one best reveals the underlying growth mechanisms? It's clear that comparing the well fitted network properties mentioned above is not sufficient to identify the best-fitting model. It needs a discriminative method for network comparison to evaluate the fit of the models to the data.

Recent studies of structural comparison for PPI networks show that the comparison methods based on local structural properties, such as graphlet counts [26]–[28] or subgraph census [29], have a strong power in discriminating the differences between networks. However, the methods paying too much attention on local network properties may fail to distinguish some obvious global differences between two networks (see section “Evaluation Results” for detailed discussions), and they usually require a large amount of computation time and will be computationally infeasible for large networks with high average degree.

To deal with these issues, we use a fast method to compare large networks that works by comparing their characteristic curves, which are shaped by both the local and global structures of the network. First, we introduce a simple graph distance to evaluate the structural difference between two networks by comparing their curves. The graph distance can then be used to evaluate the fit of a network model to the real data. We apply this evaluation method to the *Drosophila melanogaster* PPI network [30] along with three network models, including linear preferential attachment model [19] and two biologically motivated network models [21], [22]. The evaluation results then determine which model better reproduces the topology of *Drosophila's* network. We also compare our results with that achieved by a method using subgraph census and machine learning techniques [29]. And at the same time, we examine the strengths and weaknesses of the two methods.

## Methods

In this section, we first describe a network representing method. Then we apply the method to random graphs and lattice embedded random regular graphs, and derive the expressions of their characteristic curves. For the structural comparison between large networks, we introduce a graph distance based on the curve, and apply it to the *Drosophila* PPI network to evaluate the fit of the selected models to it.

### Network Representing Method

**Figure 1:**
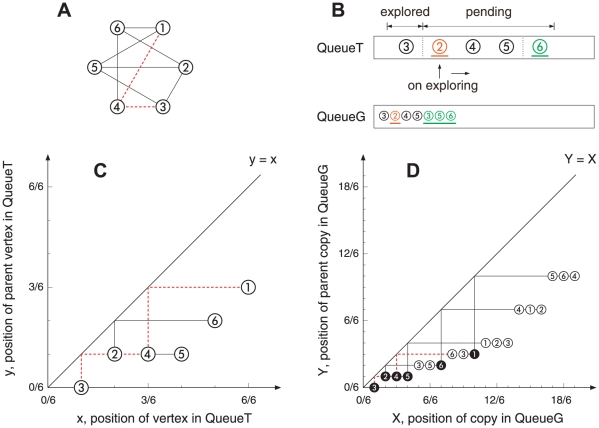
An example of the network representing method. **A:** A random 3-regular graph of six vertices, where each vertex has three neighbors randomly selected. **B:** A snapshot of the process of BFS: after vertex *3* has been explored, the pointer of QueueT moves to vertex *2*. We explore the neighbors of *2* in a random order *3*, *5*, *6*. Only untouched vertex *6* is pushed into QueueT and assigned coordinates (5/6, 2/6). To preserve all linking information of *2*, we push the copies of *3*, *5* and *6* into QueueG and assign them coordinates (5/6, 2/6), (6/6, 2/6) and (7/6, 2/6), respectively. Then the pointer moves on to *4*. **C:** BFS-tree. **D:** BFS-graph, we highlight the copies in black for their first appearances in QueueG. The line with one right angle represents an edge connecting two vertices or copies. For example, in panel **D**, polylines (2/6, 1/6)-(2/6, 2/6)-(6/6, 2/6) and (4/6, 1/6)-(4/6, 4/6)-(12/6, 4/6) represent an undirected (bidirectional) edge connecting two vertices *2* and *5*. So a vertex can see all its neighbors through a mirror placed on the line Y = X. The dotted polylines (red) represent a pathway *3* - *4* - *1*.

Consider a network of 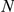 vertices and 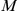 edges (the terms network/graph, vertex/node and edge/link are interchangeable in this paper). For the convenience of description, we assume that the network is undirected and connected in this section, i.e., every edge in the network is undirected and every pair of distinct vertices can be connected through some path. The proposed representing method is based on the algorithm of breadth-first search (BFS) [31], where the root vertex is selected by taking one end of a randomly chosen edge (different root selection schemes yield different outputs, the affects of root selection are discussed in details in section 3 in Supporting Information S1). One can consider the process of BFS as exploring the graph one vertex at a time in the order of first touch, first explore. At the beginning, the root vertex is labeled pending, and all other vertices are untouched. As an ongoing process (see Figure 1B), a pending vertex will be explored and all its untouched neighbors will be labeled pending and pushed into a queue named *QueueT* in a random order. Each of them is assigned a *position* 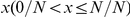 which is the ratio of its sequence in the queue to 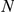, and stores 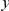, the position of its parent who brings it to the queue, i.e., who touches it at first during the process of search. Taking these two sets of positions as the coordinates 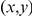 of the vertices, the search tree is mapped into a two-dimensional plane (see Figure 1C) and we refer to it as *BFS-tree*, where each edge is represented by a straight line with one right angle and parallel to each other.

Note that the BFS-tree is not a full representation of the original graph since it has lost too many edges. To get the full linking information, we now record all links of the graph during BFS. Create 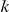 copies for each vertex of degree 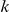, and replace each undirected edge with two opposite directed edges connecting two copies owned by the corresponding vertices. Unlike QueueT which only accepts untouched neighbors of the vertex on exploring, another queue named *QueueG* accepts the copies of all its neighbors to preserve full linking information (see Figure 1B). Meanwhile, it is similar to the vertices of QueueT that each copy of QueueG is assigned a position 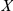 (the ratio of its order in QueueG to 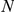) and stores 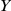 (the position of its parent copy). Thus the coordinates 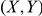 help to map a network into a two-dimensional plane (see Figure 1D) which is referred to as *BFS-graph*.

**Figure 2:**
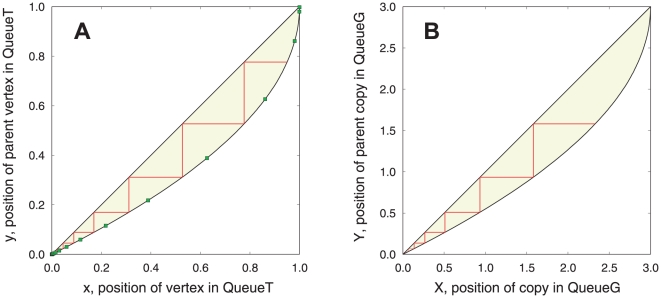
Diagrams of a random 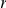 **-regular graph of size** 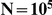 **and** 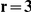. **A:** BFS-tree, where vertices are closely located around the curve 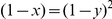. Each small square (green) represents the last vertex of its tree level of the BFS tree. **B:** BFS-graph, where copies of vertices are closely located around the curve 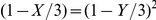. In the two diagrams, the shaded areas (yellow) represent the edges, and the polylines with right angles (red) represent a same shortest path between the root and a destination node.

Both the BFS-tree and BFS-graph are in the two-dimensional plane, and every vertex or copy can see its neighbors through a mirror placed on the line 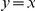 or 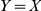. By associating vertex and edge with optical element and light beam, respectively, such a simple layout has potential applications in manufacturing large-scale optical networks. For a large network, as illustrated in Figure 2, the global picture becomes very clear where the vertices or copies line up, and automatically forms a particular curve. Since the BFS-graph holds more linking information than the BFS-tree, we here use the curve of the BFS-graph to represent the corresponding network and refer to it as the *characteristic curve*.

### Characteristic Curves

It is desirable to find the exact expressions of the characteristic curves for various networks, and see whether the curves indeed identify networks of different structures. To proceed, let us first track the states of QueueT and QueueG. During the process of BFS, network is explored one vertex at a time (can also be explored one edge at a time, the conclusions are consistent, see section 1 B in Supporting Information S1 for details). Consider a vertex 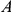 to be explored at time 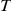 has graph degree 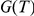, and also 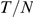 is 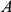
's position in QueueT. After 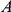 is explored at time 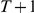, it has one parent and 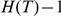 newly touched children, where 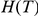 is 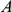
's degree on the search tree. The states of QueueT and QueueG change as follows, probing the linking information of network:
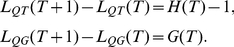
(1)where 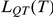 is the number of vertices that QueueT holds and 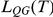 is the number of copies that QueueG holds right before exploring 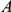 at time 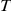. In the proposed representing method, each vertex or copy is assigned a coordinates 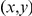 or 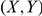 which records the positions of it and its parent. Thus, when the network is explored one vertex at a time, Eq.1 can be written as:
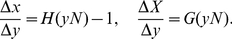
(2)where the initial values of 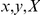 and 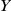 are all zeroes, and 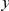 increases at a rate of 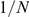 per time step. Hence, knowing the values of every vertex's graph degree 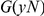, tree degree 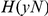 and its position 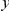 in QueueT are crucial for the derivation of the curve expressions.

We then apply this approach to two undirected networks. One is random graphs with arbitrary degree distributions, including random regular graph (RRG), Poisson-distributed random graph (PoissonRG) and power-law distributed random graph (PLRG). The other is lattice embedded random regular graph (LERRG) which is not only similar to many real-world networks, but also has practical applications. We use 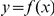 and 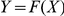 to represent the function of the tree curve and graph curve, respectively, where root vertex is in the giant component of the graph (a giant component is a connected subgraph that contains a majority of the entire graph's vertices). In general, 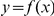 and 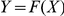 are nondecreasing and satisfy: 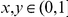, 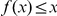, 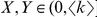 and 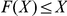, where 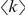 is the average degree of the graph. The smallest positive root of 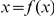 is just the size of the giant component.

#### Random Graphs with Arbitrary Degree Distributions

Suppose the degree distribution of a random network is 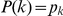, defined as the probability that a randomly chosen vertex has 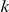 edges. Meanwhile, consider the network is obtained from the configuration model [3]: create 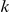 copies for each vertex of degree 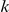, and then choose pairs of these copies uniformly at random and connect them to form the edges. Such network is a multi-graph with self-loops and multiple edges permitted. To derive the curve expressions of BFS-tree and BFS-graph for this network, as Eq. 1 shows, we should at first know the values of 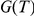 and 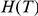 varying with 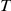.

During the process of BFS, QueueT accepts newly touched vertices one by one and assigns them positions. The term 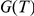 stands for the number of edges possessed by a vertex with position 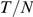. To trace the value of 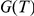 varying with 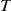, consider a situation when QueueT has accepted 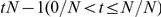 vertices and is going to accept a new one 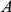. The new vertex 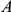 will be pushed into QueueT and assigned position 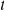, our goal is to find 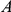
's degree 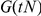.

Vertex 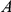 is selected from the 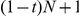 untouched vertices. Because in a random network, the copies of vertices are coupled uniformly at random, the probability of vertex 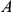 having degree 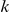 is proportional to 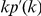, where 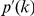 is the degree distribution of the 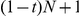 untouched vertices. The distribution 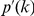 varies with 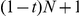 when QueueT obtains untouched vertex one by one. For the technical convenience to describe the relationship between 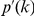 and 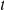, we use 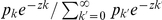 to represent 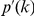, where 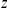 is a variable changes as a function of 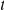: 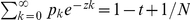. Let
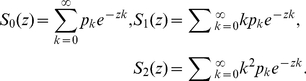
(3)where 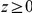 (note that 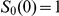 and 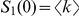, which is the average degree of the graph). Then we arrive at the distribution 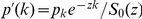, where 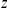 changes as a function of 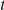 in the limit of large 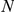 (the term 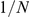 is omitted):
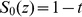
(4)


Let 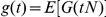 be the expected graph degree of the newly touched vertex 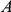. Since the probability of vertex 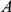 having degree 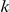 is proportional to 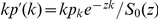, we can write:
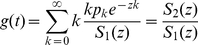
(5)


Next, we trace the value of the tree degree 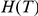. Suppose 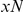 vertices have been touched before exploring a vertex 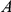 with position 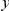. In the limit of large 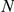, the expected number of untouched vertices that 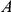 will meet through its 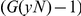 edges (except one edge connecting its parent) is:
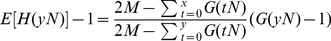
(6)where 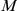 is the total number of edges, see section 1 A in Supporting Information S1 for the detailed explanation of this equation. This equation is also valid for random graphs with extremely dense edges (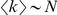), which have numerous self-loops and multi-edges (see section 1 B in Supporting Information S1 for details).

In the limit of large 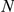, we use a mean-field approximation where 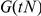 and 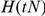 are represented by their expectations 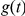 and 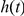, respectively. Substituting Eqs. 2 and 5 into Eq. 6 and associating it with Eqs.3 and 4, the curve function 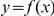 of BFS-tree satisfies (see section 1 C in Supporting Information S1 for the detailed derivation):
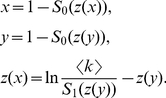
(7)where 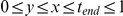, 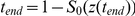. 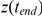 is the smallest positive root of 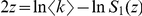. Note that 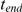 is simply the size of the giant component of the graph, which is consistent with the size derived in different forms by Molloy and Reed [32] and Newman *et al.* [33] for random graphs with arbitrary degree distributions.

From Eqs.3 and 4, we get 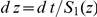, substituting this into Eqs. 2 and 5, the curve function 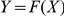 of the BFS-graph satisfies:
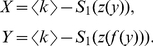
(8)where 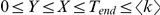, 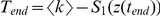. As mentioned above, 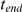 is the size of the giant component and 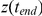 is the smallest positive root of 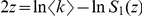. When 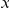 reaches 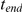, the BFS explored all vertices in the giant component and the mapping comes to the end (we here only consider the curves of the giant component since it retains the significant structural features of the graph).

As examples, we now introduce three commonly studied graphs.

**Figure 3:**
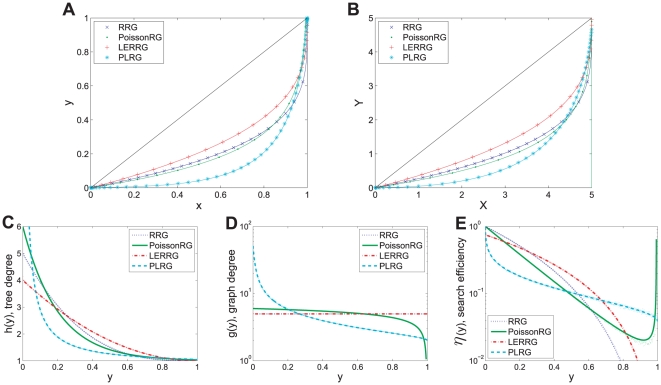
BFS-trees, BFS-graphs and auxiliary views of four example networks. Random regular graph (RRG, 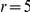), Poisson-distributed random graph (PoissonRG, average degree 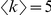), LERRG (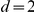, 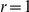, 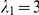) and power-law distributed random graph (PLRG, 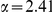, 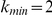, 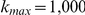, 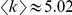) with edges not shown. In BFS-trees (panel **A**) and BFS-graphs (panel **B**), each solid line represents the vertices or copies resulted from one run of BFS on the associated network of size 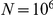, and the dots are the theoretical values. **C:** 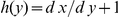, the expected tree degree of a vertex on BFS tree varies with its position 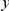 in QueueT. **D:** 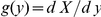, the expected graph degree. **E:** Here the expected search efficiency 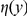 is defined as 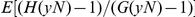 measuring the efficiency of a vertex exploring new ones through its edges (the 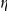 of vertices with one degree are set to zero). In panels **C–E**, the tiny dots are sampled uniformly from simulated results averaged over 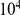 runs of BFS on the associated networks, and the lines are the analytic results.

1. *Random* 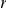 *-regular graphs*. In a graph of this kind, each vertex has a fixed degree 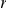, 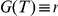. The curve functions are:
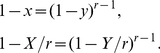
(9)where 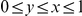, 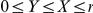, and 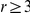 which implies that the graph is connected with high probability [34], [35].
2. *Poisson-distributed random graphs*. This is one of the best studied graph models [34], and is also known as Erdös-Rényi random graph that has a Poisson degree distribution in the limit of large graph size, as given by 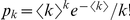. The curve functions are (see section 1 D in Supporting Information S1 for the detailed derivation):
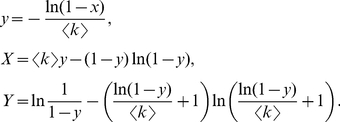
(10)where 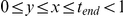, and 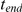 is the smallest positive root of 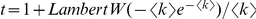. 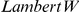 is Lambert's function, defined as 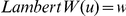 where 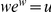.
3. *Power-law distributed random graphs*. It was found that a wide range of real networks, such as the Internet and science collaboration graph, display power-law degree distributions, also known as scale-free networks [1]. In Figure 3, we only consider a random graph possessing a power-law degree distribution given by
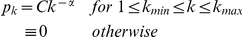
where 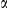 is a constant and 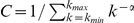. 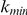 and 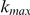 are the minimal and maximal degree of the graph, respectively. The curve expressions are the same as Eqs.7 and 8.

#### Lattice Embedded Random Regular Graphs

A graph of this type is formed from a superposition of an 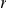-RRG and a 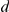-dimensional finite lattice with periodic boundary conditions, i.e., each vertex has 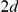 nearest lattice neighbors and 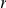 long-range neighbors chosen uniformly at random from the lattice. This is similar to the small-world model proposed by Watts and Strogatz [20], in which there are many local links and a few long-range links connecting local clusters together. These links lead to both small path lengths and high clustering called small-world property and have been observed in a wide range of real-world networks, such as the collaboration graph of film actors and the power grid. Moreover, the LERRG is not only similar to a number of real-world networks, it also has practical applications. For example, Korniss *et al.* [36] and Guclu *et al.* [37] found that two typical graphs of LERRGs have remarkable advantages in constructing a parallel discrete-event simulation scheme since the processing elements can carry out the tasks distributed on them at a nonzero, near-uniform rate without requiring global synchronization.

In the LERRG, 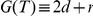, and in the limit of large network size 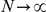, 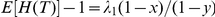, where 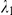 is the largest real root of 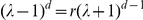 (see section 2 in Supporting Information S1 for the detailed derivation). In association with Eq. 2, the curve functions are:
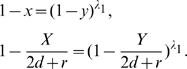
(11)Interestingly, they have a similar form as that of RRGs (Eq.9), and are consistent with Eq.9 when 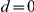.

### Graph Distance

Each of the example networks studied above corresponds to a particular curve. We here use the curve as a discriminating feature for network comparison. To evaluate the structural difference between two networks, we describe a simple graph distance 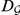 by comparing their curves
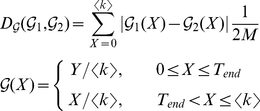
(12)where 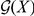 represents the characteristic curve. 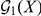 and 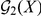 stand for the curves of a pair of graphs to be compared. The distance 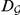 is simply the area between the two curves. Note that Onnela et al. define a graph distance based on mesoscopic response function in a similar fashion and performs well for network taxonomy [17]. Because the BFS-graph holds more linking information than the BFS-tree, we chose the curve of the BFS-graph to calculate the difference. The two-tuple 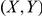 is the coordinates of vertex's copy in the BFS-graph, and 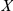 increases at a rate of 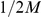, where 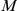 is the total number of edges in the graph. To align two graphs with different average degrees 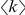, we assign 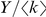 to 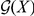 until 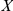 reaches 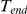, that is, until the BFS has explored all vertices in the giant component. To ease the calculation of the distance 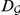 between two graphs with different sizes of the giant components, we assign 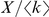 to 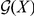 when the value of 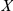 exceeds 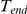. We only consider the giant component since it retains the significant structural features of the graph. For graphs which consist of small isolated groups of connected vertices, that is, whose giant components are too small (e.g., 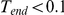) to represent the significant structural features of the entire graphs, the distance 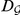 is not suitable to measure the structural difference for them.

As an example, we use 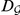 to evaluate the differences between the four example graphs in Figure 3. If we take RRG as the center graph, PoissonRG is the most similar graph with 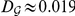 from the RRG. The LERRG is the second similar with 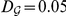 and PLRG is the most different with 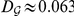. The results agree with the common understanding of the four types of graphs.

### Data Set

We use a protein-protein interaction data derived from *Drosophila melanogaster* based on yeast two-hybrid screening [30]. A PPI network can be constructed from the data by taking proteins as vertices, and observed interactions between proteins as undirected edges. The degree or connectivity of a protein is defined as the number of its interaction partners. Because the data has numerous false positives, Giot *et al.* [30] assign each interaction a confidence score 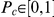, measuring how likely the interaction occurs *in vivo*. To exclude unlikely interactions, they suggest a confidence threshold 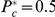. An edge appears only if its confidence score 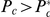. We also present results for a higher threshold 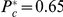 which is suggested by Middendorf *et al.* in ref. [29], and 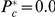 which includes all interactions observed. After removing the multiple edges and self-loops from the network [38] and eliminating isolated vertices, the resulting networks consist of 3,279/4,508/6,823 vertices and 2,728/4,569/19,630 edges for 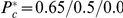, respectively.

### Network Models

We select three network models and compare their generated networks with that of the *Drosophila* to determine which model better describes the evolutionary processes of the *Drosophila*. The first two models are biologically motivated, and have been argued as the best two models to reproduce the *Drosophila* network among seven candidate models [29] including the linear preferential attachment model and the small-world model. The last one is the linear preferential attachment model. All the three models start with a small seed graph and grow the network one vertex at a time following these steps:

#### Duplication-mutation-complementation model (DMC)

The model proposes a gene duplication followed by mutations (divergence) which preserve functional complementarity [22]. At each time step, a new vertex 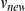 is added. It then chooses an existing vertex 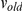 at random, and copies all links of 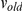, i.e., places edges between 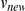 and all neighbors of 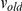. For each pair of their links connected to a same neighbor 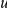, one randomly selects one of the two links (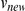, 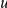) or (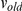, 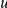) and deletes it with a probability 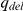. It ensures that if one of the duplicate genes loses one of its functions (links), the other preserves the same function (the link to the same neighbor). The duplicate pair 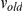 and 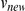 are themselves connected with a probability 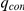, representing an interaction of a protein with its own copy. The parameters 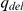 and 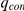 are sampled uniformly in 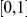.

#### Duplication-mutation using random mutations model (DMR)

The model has a different duplication algorithm from that of the DMC. It emphasizes the creation of new advantageous functions by random mutations in gene and neglects possible interactions between duplicate pairs [21]. At each time step, a newly added vertex 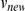 chooses an existing vertex 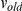 at random, and copies all links of 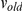. For each link of 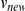 inherited from 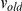, one deletes it with a probability 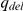. New links can be created between 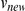 and any other existing vertices with a probability 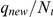, where 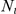 is the total number of existing vertices, introducing new viable interactions between proteins. The parameters 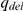 and 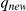 are sampled uniformly in 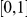.

#### Linear preferential attachment model (LPA)

At each time step, a newly added vertex preferentially attaches to existing vertices with probabilities proportional to their degrees [19]. This simple probabilistic model can give rise to scale-free degree distribution which is one of the most important features that many real-world networks exhibit, including the PPI networks.

### Network Classification Method

Given a network 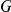 and a set of network classes, a network classifier should find which class the network 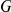 belongs to. The graph distance (Eq.12) can be used to design a simple and efficient network classifier. Consider a given set of network classes which is composed by network instances, that is, each class possesses a number of networks. If the given set of network classes is composed by network models, we generate a certain amount of network instances for each class. For each of the network classes, the classifier calculates the graph distance 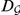 between 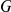 and every network in this class. We simply use the median graph distance 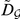 to represent the distance between 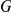 and the class, where the median graph distance is a value separating the closer half from the farther half. Finally, the classifier obtains all the median graph distances and classifies 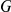 as the class which has the minimal 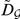 from 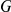.

To validate the proposed classification method, we use four network models, including the DMC, DMR, LPA and PoissonRG, by following steps. First, generate 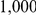 instances for each of the four models and obtain their graph curves. Second, build a network 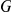 by using one of the four models, and calculate the graph distance between 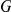 and the 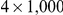 graphs generated in the first step. Classify 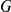 as the class which has the minimal 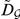 from 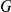. Repeat the second step 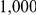 times for each of the four models, and we obtain a classification accuracy table at last (see Table 1).

**Table 1:** Classification accuracy (%) for four network models.

	Classification			
Original	DMC	DMR	LPA	PoissonRG
DMC	99.0	1.0	0.0	0.0
DMR	3.4	95.7	0.0	0.9
LPA	0.0	0.0	100.0	0.0
PoissonRG	0.0	0.0	0.0	100.0

The (i, j) entry is the probability of classifying class j given that the original class is i. The networks built by models are based on the size of the *Drosophila* protein network with a confidence threshold of 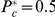.

The overall classification accuracy is high, around 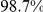, and most of these networks can find back their generative models. Classification errors among DMC, DMR, and PoissonRG networks are due to equivalence of the models in specific parameter regimes and correspondingly show overlaps. For example, when the growth parameter 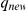 of a DMR network approximates to zero, the growth of such a network is dominated by the duplication mechanisms, which is similar to that of the DMC model. Therefore, a small fraction of DMR networks are classified as DMC.

To test the robustness of our classification method against noise, we carried out a sensitivity analysis by perturbing the structure of the original networks by using two kinds of edge random mechanisms [17], [29]. The first is to replace some percentage of original edges in the network by random ones (noise1), and the second is to randomly rewire some percentage of edges while maintaining the degree distribution of the original network (noise2). The numerical results show that the classification performs well for small and intermediate amounts of the noises on the DMC, DMR and LPA networks (see section 4 in Supporting Information S1 for details). Meanwhile, the robustness again the second noise is better than the first one since the second noise maintains the degree distribution of the original network.

## Results and Discussion

### Properties of Graph Curves

As an example shown in Figure 3, the characteristic curves coupled with auxiliary views identify networks of different topologies and reflect several local and global structural features. Among the four example networks with close average degree 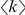, PLRG is the most special because it has an inhomogeneous degree distribution, where a small fraction of vertices (hubs) are richly connected while many other vertices are not. At an early stage of BFS on PLRG, a small fraction of vertices with high degree are firstly touched. They explore the majority of vertices and leave few opportunities for latter vertices to touch new ones. The 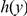, 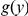 and 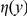 of PLRG decline with 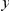 much faster than those of the other three homogeneous networks, in which the vertices have approximately the same number of edges. Such decline of LERRG is the slowest due to its high local clustering, where 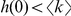 and 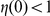. The two homogeneous random graphs RRG and PoissonRG are the most similar.

**Figure 4:**
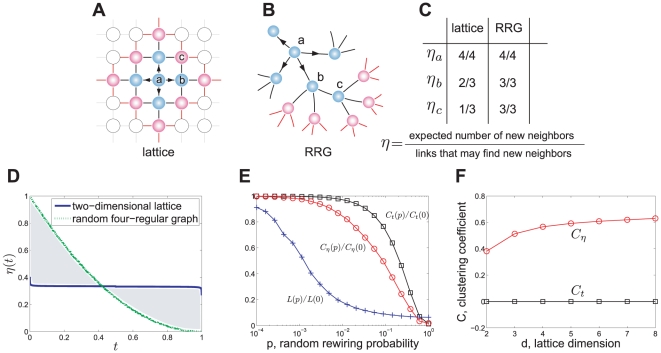
Measure the degree of local clustering. **A:** The first few steps of a BFS on a two-dimensional lattice. The blue, pink and white vertices stand for the explored, pending and untouched vertices, respectively. **B:** A BFS on a 4-RRG. **C:** Search efficiency. **D:** Search efficiencies of a network (lattice) with its random counterpart (RRG) vary with the vertices' position 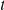 in QueueT. **E:** Average shortest path length 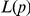, average clustering coefficient 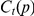 and 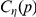 vary with the random rewiring probability 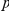 for a family of small-world networks, where the 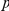 transforms a regular ring lattice (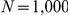 and each vertex has 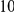 nearest neighbors) to random graphs from 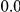 to 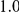. Each data point is averaged over 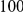 random realizations of the rewiring process, and have been normalized by the values 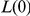, 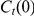 and 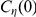 of the regular lattice. **F:** Clustering coefficient 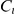 and 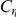 vary with 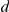, the dimension of regular lattice. Each data point is averaged over 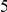 realizations of lattices with network size 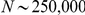.

Now we turn to characterize the structure of local clustering by the use of search efficiency 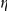. It is known that a highly clustered group of vertices has more links between them than expected by chance. A simple effect of such a structure related to BFS is that, the search explores many links but harvests less new vertices (see an example in Figure 4 A). In contrast, the search on a random graph gets more new vertices with the number close to the links explored (see Figure 4 B). Therefore, the search efficiency 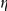 of a vertex in a lattice is smaller than that in a random graph at the first stage of search process.

Furthermore, observe that the search efficiency 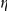 of a lattice or an LERRG is lower than that of its random counterpart (a random network with the same degree distribution allowing self-loops and multiple edges, here it is an RRG) at the early stage of the search process, but becomes larger than its counterpart later (see Figures 3 E and 4 D). That is, although the search process catches less new vertices in a clustered network than its random counterpart at first, it still has chance to meet new ones much later for its local clustering structure.

Guided by these observations, we conjecture that the larger the difference of 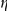 between a network 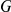 with its random counterpart 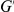, the higher the degree of local clustering of 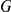 is. We then use a relative difference of 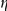 between 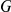 and 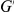 to measure the degree of local clustering of 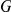:
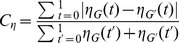
(13)which is simply the area between two 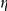 variation curves of 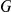 and 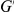 (see Figure 4 D for an example, corresponds to the shaded area between two curves), normalized by the sum of their areas.

To validate this measure, we apply it to small-world networks [20] and regular lattices which are known to have high local clustering, finding that the measure performs similar to the average clustering coefficient for small-world networks (see Figure 4 E), and can catch the local clustering of lattice network where its average clustering coefficient equals to zero (see Figure 4 F).

Next, we consider a series of characteristic curves:
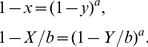
(14)where 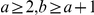 and 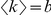. Both RRGs and LERRGs fall into this category (Eqs.9 and 11) though they are very different since the latter have high local clustering (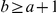) but the former have none (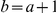).

Eq. 14 can illustrate what the associated networks look like under a single-source, all-destination traceroute sampling. In the study of Internet mapping, traceroute sampling is widely used to infer the topology of the Internet, typically by collecting paths from a small number of sources to a large number of destinations through the network. However, Lakhina *et al.* [39], Petermann and De Los Rios [40] independently showed that traceroute sampling can significantly bias the observed degree distribution since it only samples a fraction of links. In particular, they found that the sampled subgraphs have power-law degree distributions while the substrate networks are Poisson distributed. Later, Clauset and Moore [15] presented an analytical approach to derive the power law observed in ref. [39]. Achlioptas *et al.* [16] and Dall'Asta *et al.* [41] studied the bias of traceroute sampling analytically and systematically for random graphs. Interestingly, Achlioptas *et al.* found that RRGs also have apparent power laws under traceroute sampling.

**Figure 5:**
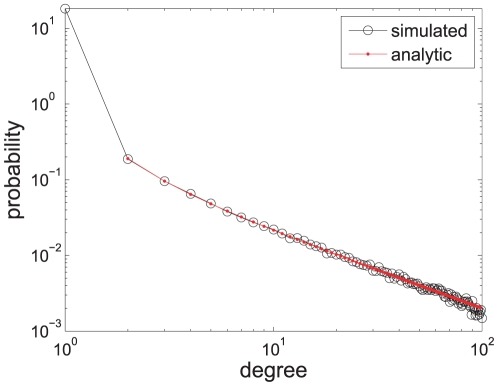
The degree distribution of a BFS tree in a LERRG (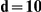, 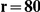). The power-law behavior 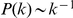 extends up to a cutoff at degree 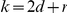. The hollow dots are results from one numerical simulation on a network of size 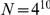, and the solid dots are our analytic results.

Here we find that even a LERRG, which is small-world-like and homogeneous, appears to have a power-law degree distribution 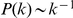 under traceroute sampling (see Figure 5). Since this sampling essentially generates a BFS tree under the common assumption that Internet routing protocols approximate shortest paths [15], [16], we turn to calculate the degree distribution of the BFS-tree. Use 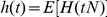 to represent the expected tree degree of a vertex with position 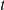 in QueueT. Eqs. 2 and 14 give
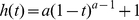
(15)Since 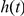 is a monotonic decreasing function, a rough estimate of the tree degree's density 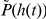 can be given by only considering the expected tree degrees during the search process
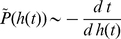
(16)substituting from Eq. 15 and letting 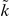 be the tree degree give
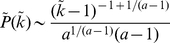
(17)where 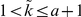. In the limit of large 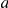, 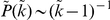. For RRGs with 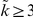, this approximate result agrees with a more rigorous one derived by different means by Achlioptas *et al.* [16]. Furthermore, our result is valid not only for RRGs but also for other networks described in Eq.14, including LERRGs. Therefore, even a LERRG, which is small-world-like and homogeneous, displays a power-law degree distribution 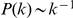 (in the limit of large 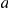) under traceroute sampling.

### Evaluation Results

**Figure 6:**
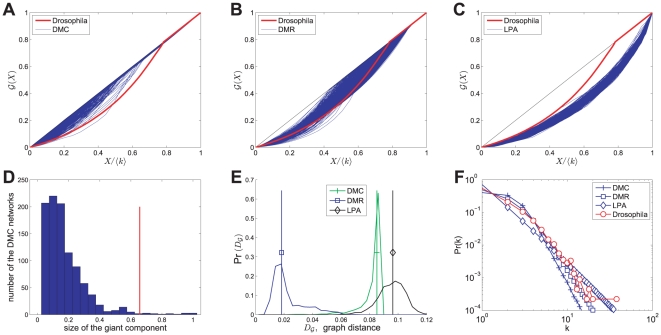
Comparisons between the model networks and the *Drosophila* PPI network for 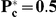. **A–C:** BFS-graphs. In each diagram, the thick red curve represents *Drosophila's* network, and the thin blue curves represent the 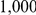 generated model networks. **D:** The size distribution of the giant components of the 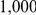 DMC networks. In *Drosophila's* network, 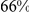 of the vertices are in the giant component (red vertical bar). **E:** Graph distance distributions. Each vertical bar represents a median graph distance 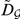 which is a value separating the closer half from the farther half to the center graph, i.e., the *Drosophila* network. **F:** Degree distributions. Each distribution of the three models is averaged over the 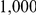 generated networks. Although their degree distributions are similar to that of the *Drosophila*, their curves vary widely.

**Figure 7:**
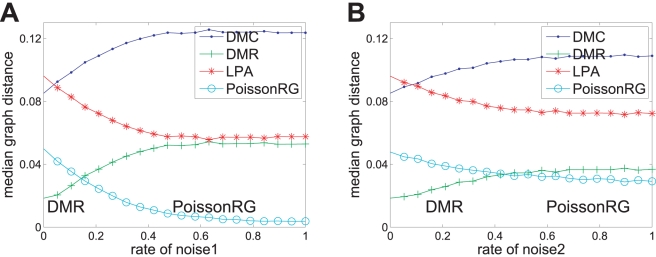
Robustness test against noises for *Drosophila* PPI network (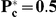). A fraction of edges in *Drosophila* network are replaced by random ones (noise1, panel **A**) or randomly rewired while maintaining the degree distribution of the original network (noise2, panel **B**). Classify the noised network as one of the four classes which has the closest median graph distance. Each data point is averaged over 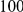 different realizations of the randomization procedure. As validation, the networks are confidently classified as a PoissonRG with the increasing of noise1.

Comparing large networks by their graph curves gives an intuitive understanding of the topological differences between the networks of *Drosophila* and each of the three models (see Figure 6, Table 2, and section 5 in Supporting Information S1 for more details). The results suggest that the DMR model better reproduces the topology of *Drosophila*'s network than the DMC and LPA for high confidence thresholds 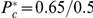. To test the robustness of this result, we artificially introduce two kinds of noises into the original *Drosophila* network (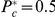), finding that the result still holds for small and intermediate amounts of the noises (see Figure 7).

**Table 2:** Median graph distance 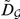 between the model networks and the *Drosophila* PPI network for different confidence thresholds 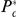, and the model with the minimal distance wins.

	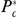 = 0.65		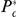 = 0.5		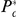 = 0.0	
Rank	Model	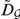	Model	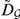	Model	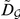
1	DMR	0.0162	DMR	0.0183	LPA	0.0351
2	LPA	0.0230	DMC	0.0851	DMR	0.0651
3	DMC	0.0310^a^	LPA	0.0963	DMC	0.2181

a For 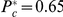, the DMC network consists of small isolated groups of connected vertices. Its giant component is too small (only around 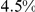 of the vertices are in the giant component, nine times smaller than that of *Drosophila*, 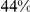) to represent the significant structural features of the entire graph. Though we give the distance value, the graph distance is not suitable for this case.

For the DMC networks, their characteristic curves are far from that of the *Drosophila*, a result which indicates that the structures of the DMC networks are very different from that of the *Drosophila*. This result is completely opposite to the result achieved by a method based on subgraph census [29], which suggests that the DMC best reproduces *Drosophila*'s network among seven candidate models, including the DMR and LPA (see Table 3).

**Table 3:** The *Drosophila* network is classified as a DMC network over DMR and LPA by a network classification method based on subgraph census [29].

	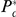 = 0.65	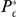 = 0.5
Rank	Model	Model
1	DMC	DMC
2	DMR	DMR
3	LPA	LPA

These contradictory results are due to the different angles from which subgraph census and BFS-graph characterize the structure of a network, where the former focuses on the substructures of the network, while the latter cares about a global view of the network. Because subgraph census counts every occurrence of a set of small subgraphs in the network, it's clear that the census can reveal more local network properties than the BFS-graph. However, subgraph census so deeply concerns the local network properties that it may fail to distinguish some obvious structural differences between two networks. The most obvious difference between the DMC and *Drosophila* is that the size of the giant component of the DMC network is much smaller than that of the *Drosophila* for high confidence thresholds 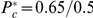, where the former is around 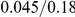 (i.e., 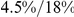 of the nodes are in the giant component), while the latter is more than nine/three times larger, 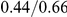 (see Figure 6 D, and section 5 in Supporting Information S1). For the higher confidence threshold 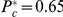, the DMC network consists of small isolated groups of connected vertices, a structure which is very different from that of the *Drosophila*. This failure of subgraph census implies that although the census knows every occurrence of the particular subgraphs in the network, it lacks a general assembly drawing of how these amounts of subgraphs are assembled into the original large network. The same amount of subgraphs may form a network different from the original network, resembling using the same building blocks to construct different buildings.

On the other hand, the BFS-graph presents a global view of the network by assembling the vertices one by one, which reflects a complementary aspect of the network to that reflected by the degree distribution and subgraph census. The degree distribution counts the degrees of all vertices and shows their distribution. Similarly, subgraph census counts the occurrences of a set of small subgraphs and shows their distribution. The two clearly know the amounts of the building blocks, but lack a general assembly drawing of how to assemble them into the original network. In contrast, the BFS-graph possesses the assembling information of the network through BFS, which strings up the vertices one by one from the bottom up, and at last, gives a global view of the network. Thus, the structural information reflected by BFS-graph and subgraph census complement each other. Applying both of them can provide a more comprehensive understanding of the network structure, which will improve the accuracy of the structural comparison.

Except for the DMC, the two methods based on BFS-graph and subgraph census agree well on the DMR and LPA, that is, the DMR better reproduces the topology of the *Drosophila* network than the LPA for 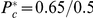. It is worth noting that the method based on BFS-graph (with time complexity 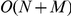) is fast for large networks with high average degree 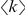, for which the subgraph census (with time complexity at least 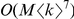) may be computationally infeasible. For example, subgraph census will cost a great deal of time for the *Drosophila* network when it includes all interactions observed (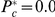), which has many more vertices and edges than that for 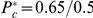. But the BFS-graph can quickly figure out the differences between the *Drosophila* network and the networks generated by the models (see section 5 in Supporting Information S1 for details). It shows that the fits of the three models to the data are relatively poor for 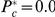 (see Table 2), a result probably due to the presence of strong additional noise in the data when including low confidence value interactions.

In summary, none of the three models is simultaneously ranked as the best by both the methods based on BFS-graph and subgraph census, implying that there is still room for improvement for these models. The DMC gets a higher rank than the DMR and LPA when using subgraph census, a result indicating that the gene duplications that preserve functional complementarity and facilitate the connections between duplicate pairs are good at reproducing the substructures of *Drosophila*'s network. When using the BFS-graph, the DMR has a closer graph distance to *Drosophila*'s network than those of the other two for high confidence thresholds 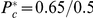, a result showing that the gene mutations that create new interactions between proteins are important for keeping the global connectivity of the PPI networks. These results suggest that a model integrating several mechanisms might be able to fit the *Drosophila* PPI network more accurately.

### Conclusions

We have presented a mesoscopic description of large networks which associates networks with a set of curves. Specific examples show that the curves can reflect a number of structural features commonly shared by a series of networks. Moreover, the curve can be used to classify networks and evaluate the fit of network models to real-world networks. After evaluating the fit of three network models to the *Drosophila* protein interaction network, we found that the model DMR better reproduces the topology of the *Drosophila* network than the DMC and LPA, although there is still room to improve the three models. We also compared our evaluation method and results with that of Middendorf *et al.*'s in ref. [29], where they identify the best-fitting model based on subgraph census, and found that the structural information reflected by characteristic curve and subgraph census complement each other. Applying the two together can provide a more comprehensive understanding of the network structure, which will improve the accuracy of the structural comparisons and model evaluations.

Using the characteristic curve, we preliminarily investigated the network properties and the fit of network models. Our further work will include the relationship study between the network structure and the curves, conditions that make this relationship one-to-one and the general algorithm (if there is one) that could recover the networks from the characteristic curves. With this algorithm and the well designed curves or functions one could generate networks with required topological features. The network describing method, in essence, utilized the process of BFS and depicted its trace on the network to capture network structure. Other processes such as random walks may also be useful to develop new approaches and applications including network characterization, comparison, classification, modeling and model evaluation.

## Supporting Information

Supporting Information S1 Supporting Information S1 includes the detailed derivations of characteristic curves of random graphs and LERRGs, discussion on the effects of root selection, robustness test of the network classification method and more network comparison results.
(PDF)
