# Supplementary material for: A Curve Shaped Description of Large Networks, with an Application to the Evaluation of Network Models
Source: PLoS One. 2011 May 17;6(5):e19784. doi: 10.1371/journal.pone.0019784 (PMC3096638; doi:10.1371/journal.pone.0019784)
Supplement: Supporting Information S1 — includes the detailed derivations of characteristic curves of random graphs and LERRGs, discussion on the effects of root selection, robustness test of the network classification method and more network comparison results. (PDF) [file pone.0019784.s001.pdf]

Supporting Information of "A Curve Shaped Description of Large Networks, with an Application to the Evaluation of Network Models"

Contents

1. Random Networks with Arbitrary Degree Distributions 1
A. Explanation of Eq. 6 based on exploring the network vertex by vertex 1
B. Explanation of Eq. 6 based on exploring the network edge by edge (stub by stub) 2
C. Derivation of Eq. 7 for random graphs with arbitrary degree distributions 3
D. Derivation of Eq. 10 for Poisson-distributed random graphs 5
E. Power-law distributed random graphs 5

2. Derivation of Eq. 11 for Lattice Embedded Random Regular Graphs (LERRGs) 6
A. The contributions of long-range neighbors 7
B. The contributions of local lattice neighbors 7
1. Lattice neighbors on a d-dimensional lattice 7
2. Lattice neighbors on a LERRG 9
C. The combined contributions of the random and lattice edges 12

3. Root Selection Effects 17

4. Test the Robustness of Network Classification Method 22

5. Network Comparison Results 24

References 26

1. RANDOM NETWORKS WITH ARBITRARY DEGREE DISTRIBUTIONS

A. Explanation of Eq. 6 based on exploring the network vertex by vertex

Suppose a network has N vertices and M edges. In Fig. S1, at time t', vertex A is firstly touched by its parent C and is pushed at the end of QueueT with position y. After that, the process of exploring is going on till time t, all the vertices between C and A in QueueT are explored, and there are xN vertices have been touched before we explore A. During this period, as illustrated in Fig. S1, one part of the *stubs* belonging to the vertices between C and A meet the vertices touched before A. While the other part of their stubs meet the vertices touched after A and including A, that is, this part of stubs have chance to meet the stubs of A, we denote the number of this part of stubs by E'.

When vertex A is on exploring at time t, it also has chance to meet the vertices untouched or touched after A (gray part of QueueT at time t in Fig. S1, including A itself). Use E_{x1} and

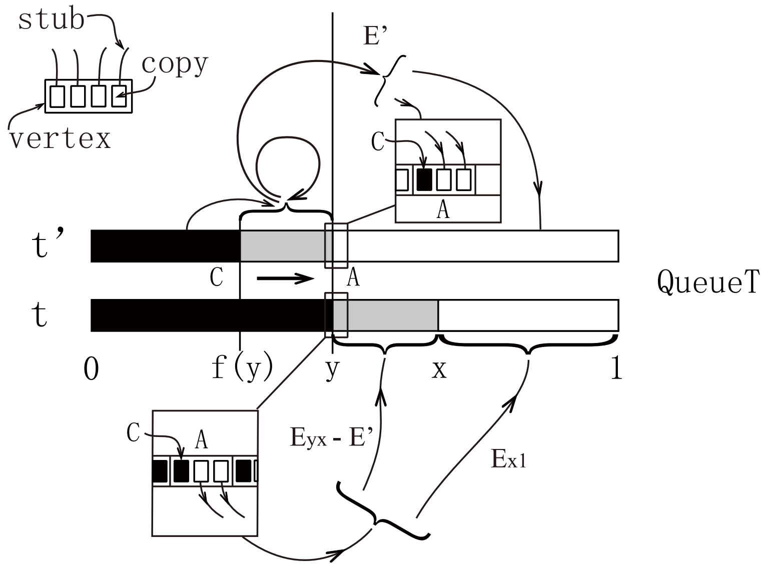

FIG. S1: Pictorial explanation of Eq. 6 in the main text based on exploring the network vertex by vertex. At a given time, the black part of QueueT are the vertices that have been explored, the gray part are the pending vertices that have been touched but not explored, and the white part represents the untouched vertices though they are not in QueueT yet. One *copy* corresponds to one *stub* sticking out of the vertex to meet other stub uniformly at random, and two connected stubs form an edge.

E_{yx} to represent the number of stubs belonging to the vertices untouched and touched after A, respectively. Since at time t the E_{yx} stubs of the vertices touched after A are partially occupied by the E' stubs come from the vertices touched before A, there are $E_{yx} - E'$ stubs left which are able to meet the stubs of A.

Therefore, vertex A has $G(yN) - 1$ stubs (except one stub connecting its parent C) to meet the stubs belonging to three parts of vertices: touched before A (E' stubs), touched after A ($E_{yx} - E'$ stubs) and untouched (E_{x1} stubs). Since the stubs of vertices are coupled uniformly at random and the probability of the appearance of self-loops and multi-edges of A goes as N^{-1}, then in the limit of large N, the expected number of untouched vertices that A will meet is

$$\begin{aligned}\frac{\Delta x}{\Delta y} &= E[H(yN)] - 1 \\ &= \frac{E_{x1}}{E' + E_{yx} - E' + E_{x1}}(G(yN) - 1) \\ &= \frac{2M - \sum_{t=0}^{x} G(tN)}{2M - \sum_{t=0}^{y} G(tN)}(G(yN) - 1) \end{aligned} \quad \text{(S1)}$$

where $E[H(yN)]$ is the expected degree of A on the breadth-first search (BFS) tree, and $G(tN)$ is the graph degree of a vertex with position t in QueueT. This equation is identical to Eq. 6 in the main text.

B. Explanation of Eq. 6 based on exploring the network edge by edge (stub by stub)

The explanation of Eq. 6 based on exploring the network vertex by vertex works well if N was much larger than the maximal degree of the graph. But it is not suitable for a random network with extremely dense edges, where the average degree $\langle k \rangle \sim N$. Since there are too many self-loops and multi-edges in such a network, the edges of a same vertex have high correlation which can not be omitted. To overcome the limitation of the explanation of Eq. 6 by exploring the network vertex by vertex, here we explore the network edge by edge:

$$\Delta y = \frac{1}{G(yN) - 1}, \quad \Delta x = \frac{2M - \sum_{t=0}^{x} G(tN)}{2M - \sum_{t=0}^{y} G(tN)}. \quad \text{(S2)}$$

where Δy means searching one edge of the vertex on exploring at one step (except the edge which brought this vertex into QueueT), and Δx is the probability that the vertex on the other end of the edge is untouched in the limit of large M.

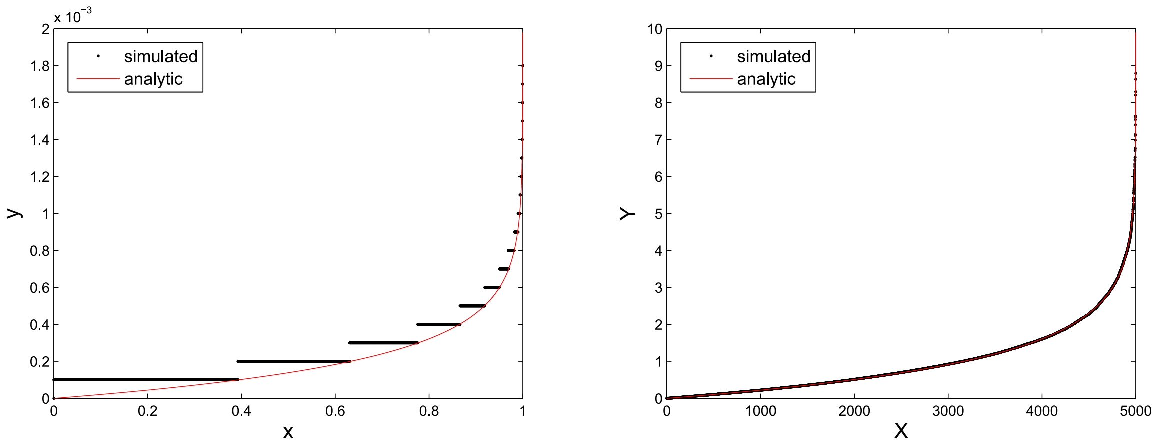

FIG. S2: BFS-tree and graph of a random r-regular graph where $r = 5,000$. The thin solid lines in red are the analytic results, following $(1 - x) = (1 - y)^{r-1}$ and $(1 - X/r) = (1 - Y/r)^{r-1}$, respectively. The dots in black are the simulated results from one run on a random regular graph of size $N = 10,000$ and $r = 5,000$.

This $\dfrac{\Delta x}{\Delta y}$ is agree with Eq. 6. Thus Eq. 6 is also valid for random networks with extremely dense edges. Fig. S2 shows an instance of random regular graph with extremely dense edges.

C. Derivation of Eq. 7 for random graphs with arbitrary degree distributions

Eq. 7 is derived from Eqs. 2, 3, 4, 5 and 6:

Eq. 2 is

$$\frac{\Delta x}{\Delta y} = H(yN) - 1, \qquad \frac{\Delta X}{\Delta y} = G(yN). \tag{S3}$$

Eq. 3 is

$$\begin{aligned} S_0(z) &= \sum_{k=0}^{\infty} p_k e^{-zk}, \\ S_1(z) &= \sum_{k=0}^{\infty} k p_k e^{-zk}, \\ S_2(z) &= \sum_{k=0}^{\infty} k^2 p_k e^{-zk}. \end{aligned} \tag{S4}$$

Eq. 4 is

$$S_0(z) = 1 - t \tag{S5}$$

Eq. 5 is

$$g(t) = \sum_{k=0}^{\infty} k \frac{kp_k e^{-zk}}{S_1(z)} = \frac{S_2(z)}{S_1(z)} \tag{S6}$$

Eq. 6 is

$$E[H(yN)] - 1 = \frac{2M - \sum_{t=0}^{x} G(tN)}{2M - \sum_{t=0}^{y} G(tN)}(G(yN) - 1) \tag{S7}$$

In the limit of large N, we use a mean-field approximation where $G(tN)$ and $H(tN)$ are represented by their expectations $g(t)$ and $h(t)$, respectively. Eq. 6 becomes the following integral equation:

$$h(y) - 1 = \frac{S_1(0) - \int_0^x g(t)\,\mathrm{d}t}{S_1(0) - \int_0^y g(t)\,\mathrm{d}t}(g(y) - 1) \tag{S8}$$

where $S_1(0) = \langle k \rangle = 2M/N$ is the average degree of graph, substituting Eqs. S3 and S6 into this equation gives integro-differential equation:

$$\frac{\mathrm{d}x}{\mathrm{d}y} = \frac{S_1(0) - \int_0^x \frac{S_2(z)}{S_1(z)}\,\mathrm{d}t}{S_1(0) - \int_0^y \frac{S_2(z)}{S_1(z)}\,\mathrm{d}t}(\frac{S_2(z(y))}{S_1(z(y))} - 1) \tag{S9}$$

From Eqs. S4 and S5, get $S_1(z)\,\mathrm{d}z = \mathrm{d}t$ and $\mathrm{d}S_1(z) = -S_2(z)\,\mathrm{d}z$, then

$$\begin{aligned}
\frac{\mathrm{d}x}{\mathrm{d}y} &= \frac{S_1(0) + \int_0^x \mathrm{d}S_1(z)}{S_1(0) + \int_0^y \mathrm{d}S_1(z)}(\frac{S_2(z(y))}{S_1(z(y))} - 1) \\
\frac{\mathrm{d}x}{S_1(z(x))} &= \frac{1}{S_1(z(y))}(\frac{S_2(z(y))}{S_1(z(y))} - 1)\,\mathrm{d}y \\
\mathrm{d}z(x) &= (\frac{S_2(z(y))}{S_1(z(y))} - 1)\,\mathrm{d}z(y) \\
\mathrm{d}z(x) &= -\mathrm{d}\ln S_1(z(y)) - \mathrm{d}z(y)
\end{aligned} \tag{S10}$$

the initial values of x, y and z are all zeroes, then

$$z(x) = \ln \frac{S_1(0)}{S_1(z(y))} - z(y).$$

along with Eq. S5, give Eq. 7

$$\begin{aligned}
x &= 1 - S_0(z(x)), \\
y &= 1 - S_0(z(y)), \\
z(x) &= \ln \frac{S_1(0)}{S_1(z(y))} - z(y).
\end{aligned} \tag{S11}$$

where $0 \le y \le x \le t_{end} \le 1$, $t_{end} = 1 - S_0(z(t_{end}))$. $z(t_{end})$ is the smallest positive root of $2z = \ln S_1(0) - \ln S_1(z)$.

D. Derivation of Eq. 10 for Poisson-distributed random graphs

The degree distribution of this graph obeys:

$$p_k = \frac{\langle k\rangle^k}{k!} e^{-\langle k\rangle}$$

Then with Eq. S5

$$\mathrm{d}t = S_1(z)\,\mathrm{d}z = \langle k\rangle e^{-z} S_0(z)\,\mathrm{d}z = \langle k\rangle e^{-z}(1-t)\,\mathrm{d}z \tag{S12}$$

the initial values of t and z are all zeros, then

$$\langle k\rangle e^{-z} = \ln(1-t) + \langle k\rangle \tag{S13}$$

with Eq. S6,

$$g(t) = S_2(z)/S_1(z) = \langle k\rangle e^{-z} + 1 = \ln(1-t) + \langle k\rangle + 1 \tag{S14}$$

substituting this equation into Eq. S3 and Eq. S8, the curve functions are:

$$\begin{aligned} y &= -\frac{\ln(1-x)}{\langle k\rangle}, \\ X &= \langle k\rangle y - (1-y)\ln(1-y), \\ Y &= \ln\frac{1}{1-y} - \left(\frac{\ln(1-y)}{\langle k\rangle} + 1\right)\ln\left(\frac{\ln(1-y)}{\langle k\rangle} + 1\right). \end{aligned} \tag{S15}$$

where $0 \leq y \leq x \leq t_{end} < 1$, and t_{end} is the smallest positive root of $t = 1 + LambertW(-\langle k\rangle e^{-\langle k\rangle})/\langle k\rangle$. $LambertW$ is Lambert's function, defined as $LambertW(u) = w$ where $we^w = u$.

E. Power-law distributed random graphs

In this text, we consider a power-law degree distribution given by

$$p_k = \begin{cases} Ck^{-\alpha} & \text{for } 1 \leq k_{min} \leq k \leq k_{max} \\ 0 & \text{otherwise} \end{cases}$$

where α is a constant and $C = 1/\sum_{k=k_{min}}^{k_{max}} k^{-\alpha}$. k_{min} and k_{max} are the minimal and maximal degree of the graph, respectively. By the means used in the main text for random graphs

with arbitrary degree distributions, let

$$\begin{aligned} S_0(z) &= \sum_{k=k_{min}}^{k_{max}} Ck^{-\alpha}e^{-zk}, \\ S_1(z) &= \sum_{k=k_{min}}^{k_{max}} Ck^{-\alpha+1}e^{-zk}, \\ S_2(z) &= \sum_{k=k_{min}}^{k_{max}} Ck^{-\alpha+2}e^{-zk}. \end{aligned} \tag{S16}$$

where z is a variable with initial value $z_0 = 0$ and suppose they are all finite. The curve functions of BFS-tree and BFS-graph of this power-law distributed random graph follow the same form of Eq. 7 and Eq. 8 in the main text:

$$\begin{aligned} x &= 1 - S_0(z(x)), \\ y &= 1 - S_0(z(y)), \\ z(x) &= \ln \frac{S_1(0)}{S_1(z(y))} - z(y), \\ X &= S_1(0) - S_1(z(y)), \\ Y &= S_1(0) - S_1(z(f(y))). \end{aligned} \tag{S17}$$

2. DERIVATION OF EQ. 11 FOR LATTICE EMBEDDED RANDOM REGULAR GRAPHS (LERRGS)

The LERRG is an uncorrelated combination of a random r-regular graph and a d-dimensional finite lattice with periodic boundary conditions, i.e., each vertex has $2d$ nearest lattice neighbors and r long-range neighbors chosen uniformly at random from the lattice. The natural numbers d and r are supposed such that $N \gg d \geq 1$, $N \gg r \geq 1$, and rN is even to ensure that the copies of vertices can be coupled randomly.

To get the curve functions of BFS-tree and graph, we should know how many untouched neighbors that a vertex on exploring will visit (see Eq. 2 or Eq. S3). There are two contributions to this number: long-range neighbors connected by random edges and local neighbors on lattice.

A. The contributions of long-range neighbors

The situation is similar to that of random graphs with arbitrary degree distributions. Suppose xN vertices have been touched before we explore a vertex A of position y. Vertex A is in one of the two cases:

(i) Vertex A was brought into QueueT by a random edge from its parent, then similar to Eq. S1, for large N, the expected number of untouched vertices that A will meet through its random edges is

$$\frac{2M - 2dN - \sum_{t=0}^{x}(G(tN) - 2d)}{2M - 2dN - \sum_{t=0}^{y}(G(tN) - 2d)}(G(yN) - 2d - 1) \tag{S18}$$

where $G(tN)$ is the graph degree of the vertex of position t in QueueT. In this graph, $2M = (2d + r)N$ and $G(tN) \equiv 2d + r$ $(t \in (0, 1])$, Eq. S18 can be written as

$$\frac{1 - x}{1 - y}(r - 1) \tag{S19}$$

(ii) Vertex A was brought into QueueT by a lattice edge, it has r random edges free and the expected number of untouched vertices that A will meet through its random edges is

$$\frac{1 - x}{1 - y}r \tag{S20}$$

B. The contributions of local lattice neighbors

To be easily understood, we consider the effect of lattice edges on a single d-dimensional lattice at first, and then turn to the LERRG.

1. *Lattice neighbors on a d-dimensional lattice*

Let a vertex O be the origin vertex of the lattice and $(x_1, \ldots, x_d)$ be the coordinates of a vertex on lattice, where $x_1, \ldots, x_d \in \mathbb{Z}$. Define the lattice distance between O and a vertex with coordinates $(x_1, \ldots, x_d)$ to be the number of lattice steps separating them: $\sum_{i=1}^{d} |x_i|$.

Denote by L_l the *type* of a vertex $l(l \in \mathbb{N})$ lattice steps away from the origin, and by N_j the *type* of a vertex with $j(j = 0, 1, \ldots, d)$ nonzero components in its coordinates.

The process of BFS starts from the origin and forms a "**crystal**" (see Fig. S3(a)). Since the search order is random, the touching order of all vertices of type L_l is random. Suppose

a vertex A of type L_{l+1} has j neighbors of type L_l, then each of these neighbors shares an equal chance $\dfrac{1}{j}$ to touch vertex A at first. For example:

$$(1,-3,0,5,-1),(2,-2,0,5,-1),$$
$$(2,-3,0,4,-1),(2,-3,0,5,0).$$
$$\Downarrow$$
$$(2,-3,0,5,-1)$$

where vertex $C(2,-3,0,5,0)$ is of type L_{10} and N_3, vertex $A(2,-3,0,5,-1)$ is of type L_{11} and N_4. Vertex C has a probability of 1/4 to touch vertex A at first among these four vertices of type L_{10}.

Now, we consider how many untouched vertices of type L_{l+1} will be visited by a vertex of type L_l on exploring.

Lemma S1 *During the process of BFS on a d-dimensional lattice, a vertex C of type* $N_j (j = 0, 1, \ldots, d)$ *will visit* $2\frac{d-j}{j+1}$ *untouched vertices of type* N_{j+1}*, and* $\frac{j}{j}$ *untouched vertices of type* N_j *on average. The total expected number of new vertices that C will visit is* $2\frac{d-j}{j+1}+1$.

Proof.

There are two cases for vertex C:

(i) Suppose vertex C is of type L_l and $N_j (j < d)$. It has $2\binom{d-j}{1}$ lattice neighbors of type L_{l+1} and N_{j+1}. Since a vertex of type L_{l+1} and N_{j+1} will be touched firstly by vertices of type L_l with equal probability $\frac{1}{j+1}$, then C will touch $2\binom{d-j}{1}\frac{1}{j+1}$ vertices of type L_{l+1} and N_{j+1} on average. On the other hand, C has j lattice neighbors of type L_{l+1} and N_j. Then the expected number of untouched vertices that C will visit is

$$2\binom{d-j}{1}\frac{1}{j+1}+\frac{j}{j}=2\frac{d-j}{j+1}+1 \tag{S21}$$

(ii) Suppose vertex C is of type L_l and N_d. Since C only has d lattice neighbors of type L_{l+1} and all of them are of type N_d, then C will visit $\frac{d}{d}=1$ untouched vertices on average. In this case, $2\frac{d-j}{j+1}+1$ is also valid.

2. Lattice neighbors on a LERRG

For the existence of long-range random edges, small crystals grow randomly all over the lattice during the process of BFS (see Fig. S3(b)). Typically, a vertex O is firstly touched by a random edge, and then with the process of search going on, O will explore a small crystal within a few lattice steps purely through lattice edges. The vertices of this crystal share a same **crystal origin** O and their **crystal coordinates** are the relative coordinates if we took O as the origin of the lattice.

As defined in subsection 2 B 1, denote by L_l the type of a vertex $l(l \in \mathbb{N})$ lattice steps away from its crystal origin, and by N_j the type of a vertex with $j(j = 0, 1, \ldots, d)$ nonzero items in its crystal coordinates.

During the process of BFS, consider a vertex A of type L_l and N_j is on exploring. The position of A in QueueT is y and there are xN vertices have been touched and pushed into QueueT (see Fig. S4). Vertex C of position $f(y)$ is the parent of A. What we want to know

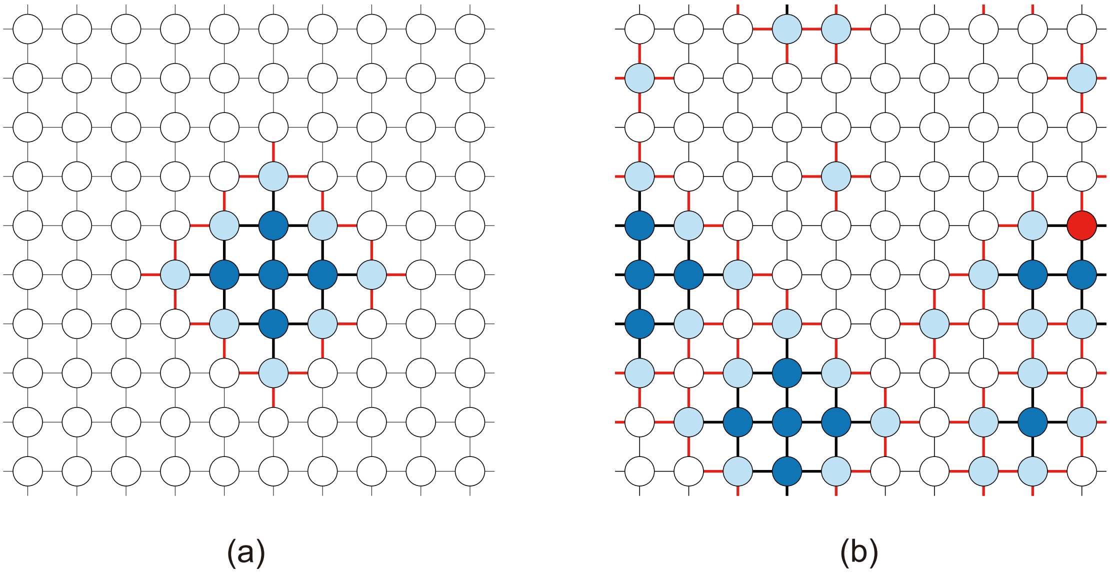

FIG. S3: Two snapshots of the process of BFS on two lattices with periodic boundary conditions. (a) A two-dimensional lattice with one "crystal". (b) A lattice embedded random regular graph of $d = 2$ and $r = 1$ with a few crystals. Here we only draw the lattice edges without disordered random edges. In both snapshots, explored vertices are in dark blue, pending vertices are in light blue, the white ones are the untouched vertices and the red one is the vertex on exploring. Red thick edges are free lattice edges.

is how many untouched vertices will be visited by A through its lattice edges.

Here we call the lattice edge connecting vertex A and a vertex of type L_{l+1} ($l+1$ lattice steps away from A's crystal origin) as **free lattice edge** of A (edges in red in Fig. S3), and denote this type of edge by FLE_j if the vertex on the other end was of type N_j. By Lemma S1, A has $2(d-j)$ and j free lattice edges of type FLE_{j+1} and FLE_j, respectively. These free lattice edges may lead us to untouched neighbors on lattice.

Lemma S2 *During the process of BFS on a LERRG of d-dimension, suppose xN vertices have been touched before we explore a vertex A of position y in QueueT. The expected number of untouched vertices that A of type L_l and N_j will visit through lattice edges is*

$$\left(2\frac{d-j}{j+1}+1\right)\frac{1-x}{1-y} \tag{S22}$$

where $2\frac{d-j}{j+1}\frac{1-x}{1-y}$ of them are of type N_{j+1}, and $\frac{1-x}{1-y}$ of them are of type N_j.

Proof.

We will detect every free lattice edge of A and calculate the probability that the vertex on the other end is untouched.

Suppose a vertex B is connected by a free lattice edge of A. For the structural feature of lattice, B is equally shared by A and A's nearby vertices of the same crystal. Suppose there are j vertices (sharing the same crystal origin with A and including A) have free lattice edges of type FLE_j connecting B (i.e., B is of type N_j). Then, among these j vertices, the probability of A touching B at first through lattice edges is $1/j$. Otherwise, B has already been touched by other $j-1$ vertices before A. We then turn to calculate the probability that B is untouched right before we explore A, in the condition that A is the first vertex touching B among those j vertices through lattice edges.

Let $\overrightarrow{AB}$ be the lattice edge from A to B, and $\overrightarrow{BA}$ be the lattice edge from B to A. $\overrightarrow{AB}$ and $\overrightarrow{BA}$ form the undirected lattice edge between A and B. Since every vertex has long-range random links and visits its neighbors in a random order, the position of B in QueueT is uncertain. But B must be one of the vertices whose lattice edges of $\overrightarrow{BA}$-direction have chance to meet A, that is, every vertex has equal chance to be B if its $\overrightarrow{BA}$-directed lattice edge has chance to meet A. Then we turn to find out the vertices whose lattice edges of $\overrightarrow{BA}$-direction have chance to meet A during the process of breadth-first search.

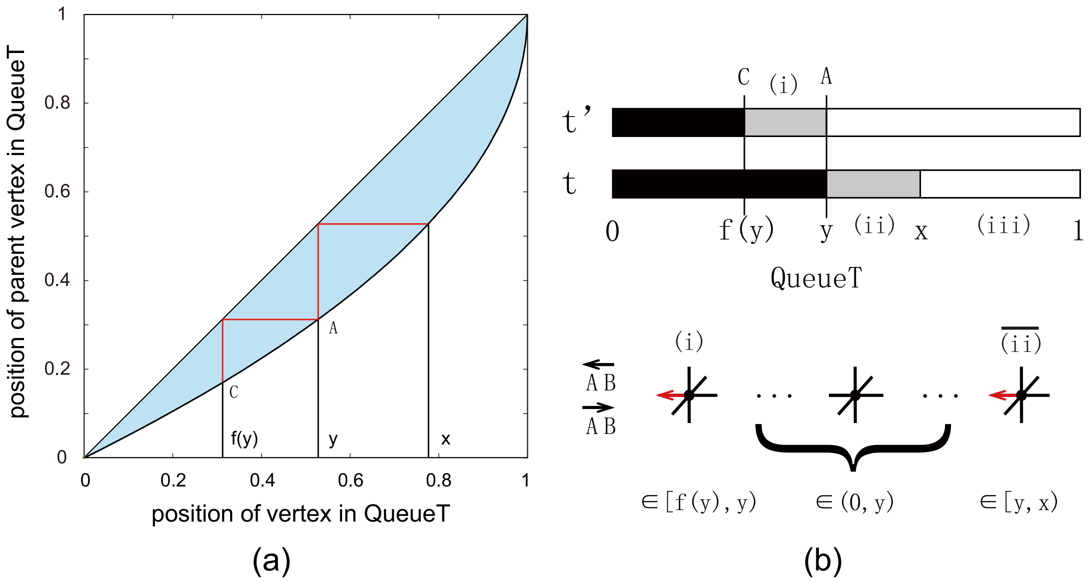

FIG. S4: Auxiliary charts for the explanation of Lemma S2. At a given time, the black part of QueueT are the vertices that have been explored, the gray part are the pending vertices and the white part represents the untouched vertices. $\overline{(ii)}$ represents the excluded vertices in case (ii) of subsection 2 B 2.

As Fig. S4 shows, from the time t' when vertex A is firstly brought into QueueT by its parent C to the time t when all pending vertices between C and A are explored, the newly touched vertices are all located between position y and x in QueueT. Since C is the first vertex that brings A into QueueT, the position of B is not in $(0, f(y))$. B is in one of the three cases as below (see Fig. S4):

(i) The position of B is in $[f(y), y)$. Every vertex located in $[f(y), y)$ has equal chance to be B except the vertex whose lattice edge of $\overrightarrow{BA}$-direction connects vertex in $(0, y)$, since it has no $\overrightarrow{BA}$-directed lattice edge left to connect vertex A. Denote by C_1 the number of vertices which have equal chance to be B in this range.

(ii) The position of B is in $[y, x)$. Every vertex located in $[y, x)$ has equal chance to be B except the vertex whose $\overrightarrow{BA}$-directed lattice edge connects vertex in $[f(y), y)$.

(iii) B has not been touched yet. Every untouched vertex has its $\overrightarrow{BA}$-directed lattice edge free and B is one of the $N(1-x)$ untouched vertices.

As Fig. S4(b) shows, when we walk from any excluded vertex in case (ii) in the direction of $\overrightarrow{BA}$ along lattice edges, we will meet one vertex of case (i). And the positions of the vertices along the lattice path between these two vertices are all in $(0, y)$. Vice versa, if we

walk from any vertex of case (i) in the direction of $\overrightarrow{AB}$ we will meet one vertex excluded in case (ii). Therefore, the number of vertices excluded in case (ii) is equal to C_1 the number of vertices of case (i). Then the number of touched vertices that have equal chance to be B is $C_1+(N(x-y)-C_1) = N(x-y)$. Vertex B is among these $N(x-y)+N(1-x) = N(1-y)$ vertices.

Therefore, the probability that B has not been touched yet before we explore A is

$$\frac{j-1}{j}0+\frac{1}{j}\frac{N(1-x)}{N(1-y)} = \frac{1}{j}\frac{1-x}{1-y} \tag{S23}$$

where B is of type N_j. Then, by Lemma S1, the expected number of untouched vertices that A of type L_l and N_j will visit through lattice edges is

$$\left(2\frac{d-j}{j+1}+1\right)\frac{1-x}{1-y} \tag{S24}$$

where $2\frac{d-j}{j+1}\frac{1-x}{1-y}$ of them are of type N_{j+1}, and $\frac{1-x}{1-y}$ of them are of type N_j.

C. The combined contributions of the random and lattice edges

Suppose a vertex A of position y has a child of position x in QueueT. Let ρ_j and ρ'_j be the probability of A and its child to be the type of $N_j(j = 0, 1, \ldots, d)$, respectively. Where $\sum_{j=0}^{d}\rho_j = 1$ and $\sum_{j=0}^{d}\rho'_j = 1$. Although there are many crystals interlaced all over the lattice during the process of BFS, as illustrated in Fig. S3(b), the type (N_j) of a vertex is determined only once by its parent who touch it at first. One vertex only has one type. For example, if a vertex was brought into QueueT by a random edge firstly, then the type of this vertex is N_0.

Considering a parent vertex of all possible types (N_j) and in association with Eqs. S19 and S20 and Lemma S2, the relationship between a vertex and its children on search tree

satisfies:

$$\begin{pmatrix} 1 & c_{d-1} & \cdots & 0 & 0 \\ 0 & 1 & \cdots & 0 & 0 \\ \vdots & \vdots & \vdots & \vdots & \vdots \\ 0 & 0 & \cdots & 1 & c_0 \\ r & r & \cdots & r & r-1 \end{pmatrix} \begin{pmatrix} \rho_d \\ \vdots \\ \rho_1 \\ \rho_0 \end{pmatrix} \frac{1-x}{1-y}$$

$$= (\sum_{j=1}^{d}(\rho_j + c_{j-1}\rho_{j-1}) + r - \rho_0) \begin{pmatrix} \rho'_d \\ \vdots \\ \rho'_1 \\ \rho'_0 \end{pmatrix} \frac{1-x}{1-y} \tag{S25}$$

where

$$\begin{aligned} c_j &= 2\frac{d-j}{j+1},\ j = 0, \ldots, d-1 \\ \rho'_j &= \frac{\rho_j + c_{j-1}\rho_{j-1}}{\sum_{j'=1}^{d}(\rho_{j'} + c_{j'-1}\rho_{j'-1}) + r - \rho_0}, \quad j = 1, \ldots, d \\ \rho'_0 &= \frac{r - \rho_0}{\sum_{j'=1}^{d}(\rho_{j'} + c_{j'-1}\rho_{j'-1}) + r - \rho_0}. \end{aligned} \tag{S26}$$

where ρ_j and ρ'_j are the probability of A and its child being the type of N_j, respectively. Therefore, in the limit of large network size, the expected number of untouched vertices that A will visit is:

$$\frac{\Delta x}{\Delta y} = (\sum_{j=1}^{d}(\rho_j + c_{j-1}\rho_{j-1}) + r - \rho_0)\frac{1-x}{1-y} \tag{S27}$$

It is difficult to calculate $(\sum_{j=1}^{d}(\rho_j + c_{j-1}\rho_{j-1}) + r - \rho_0)$ directly, we will show that it approaches the dominant eigenvalue of matrix M_{dr} and $(\rho'_d, \cdots, \rho'_1, \rho'_0)^\top$ approaches the associated eigenvector.

$$M_{dr} = \begin{pmatrix} 1 & c_{d-1} & \cdots & 0 & 0 \\ 0 & 1 & \cdots & 0 & 0 \\ \vdots & \vdots & \vdots & \vdots & \vdots \\ 0 & 0 & \cdots & 1 & c_0 \\ r & r & \cdots & r & r-1 \end{pmatrix} \tag{S28}$$

The eigenfunction of matrix M_{dr} is

$$(\lambda - 1)((\lambda - 1)^d - r(\lambda + 1)^{d-1}) = 0 \tag{S29}$$

where $r \geq 1$ and $d \geq 1$. Note that Eq. S29 has a largest real root. Denote the roots of Eq. S29 by $\lambda_1, \lambda_2, \ldots, \lambda_{d+1}$, and they satisfy:

$$|\lambda_1| > |\lambda_2| \geq |\lambda_3| \geq \cdots \geq |\lambda_{d+1}| \geq 0 \tag{S30}$$

where λ_1 is the largest real root.

Therefore, M_{dr} has $d + 1$ eigenvalues $\lambda_1, \lambda_2, \ldots, \lambda_{d+1}$ with an associated collection of linearly independent eigenvectors, and λ_1 is the dominant eigenvalue.

Refer to the power method used in finding the dominant eigenvalue of a matrix in the study of numerical analysis [1, 2], let $v_\rho^s = (\rho_d^s, \cdots, \rho_1^s, \rho_0^s)^\top$ be the vector after s steps of iterations, and v_ρ^s is initialized with $v_\rho^0 = (0, \cdots, 0, 1)^\top$ for all the vertices are of type N_0 at the beginning, they satisfy

$$\begin{pmatrix} 1 & c_{d-1} & \cdots & 0 & 0 \\ 0 & 1 & \cdots & 0 & 0 \\ \vdots & \vdots & \vdots & \vdots & \vdots \\ 0 & 0 & \cdots & 1 & c_0 \\ r & r & \cdots & r & r-1 \end{pmatrix} \begin{pmatrix} \rho_d^s \\ \vdots \\ \rho_1^s \\ \rho_0^s \end{pmatrix}$$

$$= (\sum_{j=1}^{d} (\rho_j^s + c_{j-1}\rho_{j-1}^s) + r - \rho_0^s) \begin{pmatrix} \rho_d^{s+1} \\ \vdots \\ \rho_1^{s+1} \\ \rho_0^{s+1} \end{pmatrix} \tag{S31}$$

$$\lim_{s \to \infty} (\sum_{j=1}^{d} (\rho_j^s + c_{j-1}\rho_{j-1}^s) + r - \rho_0^s) = \lambda_1 \tag{S32}$$

The rate of convergence depends on $|\lambda_2/\lambda_1|$. Table S1 shows that $(\sum_{j=1}^{d}(\rho_j^s + c_{j-1}\rho_{j-1}^s) + r - \rho_0^s)$ approaches λ_1 with high accuracy after few iterations. Each component (ρ_j) of the associated eigenvector of λ_1 represents the probability of a vertex to be a corresponding vertex type (N_j).

Therefore, in the limit of large network size, Eq. S27 can be written as

$$\frac{\mathrm{d}x}{\mathrm{d}y} = \lambda_1 \frac{1-x}{1-y} \tag{S33}$$

With Eq. S3, the curve functions of the BFS-tree and BFS-graph are

$$\begin{aligned}(1-x) &= (1-y)^{\lambda_1},\\ (1-\frac{X}{2d+r}) &= (1-\frac{Y}{2d+r})^{\lambda_1}.\end{aligned} \tag{S34}$$

where λ_1 is the largest real root of $(\lambda-1)^d = r(\lambda+1)^{d-1}$ (Eq. S29). Tables S2 and S3 show that the analytic results are well consistent with the simulated results.

TABLE S1: The minimal iteration steps s_{min} satisfying that $(\sum_{j=1}^{d}(\rho_j^s + c_{j-1}\rho_{j-1}^s) + r - \rho_0^s)$ approaches λ_1 with relative error less than 0.0001

$d\diagdown r$	1	2	3	4	5	6	7	8
1	1	1	1	1	1	1	1	1
2	2	4	4	4	4	4	4	4
3	6	5	5	5	4	3	3	3
4	7	6	6	5	5	5	5	4
5	7	6	6	6	5	5	5	5
6	9	7	6	6	6	5	5	5
7	10	8	6	6	6	6	5	5
8	10	7	7	6	6	6	6	5

Note that these functions are consistent with that of random regular graphs (Eq. 9 in the main text) when $d = 0$. Comparing LERRGs with random regular graphs, there are two interesting cases. One is the LERRGs of $d = 1$ (a ring combined with a random regular graph), where both the curve functions of BFS-tree and BFS-graph are same as that of random $(2d+r)$-regular graphs. This is due to the fact that such LERRGs are equivalent to the corresponding random regular graph which possesses a Hamiltonian circuit. The event of a random r-regular graph of $r \geq 3$ possessing a Hamiltonian circuit occurs asymptotically almost surely [3, 4]. The other is the LERRG of $d = 2$ and $r = 1$, whose curve function of BFS-tree is same as that of a random 4-regular graph, i.e., $1-x = (1-y)^3$. In other words, when we explore two vertices (except few vertices close to the root) of a same position x

TABLE S2: The dominant eigenvalue of the corresponding matrix M_{dr}, rounded to four decimal places

$d\diagdown r$	1	2	3	4	5	6	7	8
1	2.0000	3.0000	4.0000	5.0000	6.0000	7.0000	8.0000	9.0000
2	3.0000	4.2361	5.3723	6.4641	7.5311	8.5826	9.6235	10.6569
3	3.8751	5.2958	6.5511	7.7305	8.8659	9.9728	11.0600	12.1326
4	4.6786	6.2593	7.6219	8.8826	10.0833	11.2447	12.3781	13.4909
5	5.4338	7.1592	8.6204	9.9572	11.2200	12.4337	13.6123	14.7647
6	6.1534	8.0126	9.5661	10.9745	12.2964	13.5603	14.7827	15.9737
7	6.8454	8.8302	10.4706	11.9471	13.3255	14.6377	15.9023	17.1308
8	7.5149	9.6187	11.3418	12.8835	14.3160	15.6748	16.9802	18.2452

TABLE S3: The relative errors between analytic solutions and simulated results of λ_1, rounded to five decimal places. Each simulated result is averaged over 10 network realizations of size $N \sim 10^7$.

$d\diagdown r$	1	2	3	4	5	6	7	8
1	0.00021	0.00025	0.00024	0.00004	0.00008	0.00007	0.00021	0.00001
2	0.00005	0.00017	0.00006	0.00017	0.00004	0.00026	0.00010	0.00008
3	0.00018	0.00028	0.00011	0.00016	0.00013	0.00016	0.00006	0.00000
4	0.00005	0.00013	0.00007	0.00009	0.00010	0.00004	0.00018	0.00011
5	0.00006	0.00006	0.00016	0.00012	0.00002	0.00014	0.00014	0.00005
6	0.00003	0.00026	0.00019	0.00014	0.00020	0.00015	0.00006	0.00001
7	0.00016	0.00027	0.00015	0.00031	0.00009	0.00007	0.00024	0.00013
8	0.00090	0.00059	0.00009	0.00010	0.00019	0.00014	0.00004	0.00008

in both graphs, the expected numbers of new vertices that the two vertices on exploring will touch are equal, though the underlying graphs are totaly different. On the other hand, their functions of BFS-graph have a good discriminating power telling the difference between them.

3. ROOT SELECTION EFFECTS

We here examine the variances of the graph curves affected by using four different root selection schemes,
(1) Pick one end of a randomly chosen edge.
(2) Pick a randomly chosen vertex.
(3) Pick a vertex with the maximal degree.
(4) Pick a Vertex with the minimal degree (at least has one edge).
for all the networks studied in this manuscript, including random graphs, LERRG, PPI network and the related network models.

For random graphs and LERRG, the above four schemes have negligible differences since the edges reaching out from a root will touch child vertices proportional to their degree, which has a same effect of picking one end of a randomly chosen edge. The expressions of the tree and graph curves for random graphs are exact in the limit of large network size. It observes gradually diminishing of the curve difference with the increasing of vertex number in simulations, as shown in Figure S5 and Tables 3 3.

However, the curve variance of real networks or networks built by models is relatively larger, as Figure S6 shows. To quantify the variance of a set of graph curves derived from a same network, we calculate the graph distance of each graph curve to a center curve which is simply the average of these curves. Denote the curve difference by $\Delta D_{\mathcal{G}}$, we use its average value $\overline{\Delta D_{\mathcal{G}}}$, which an average over all graph curves in the set, to represent the curve variance caused by the choosing of root and the processing of BFS.

Numerical results show that the $\overline{\Delta D_{\mathcal{G}}}$ of random graphs are small and decrease with a larger network size (see Table 3) under the four different root selection schemes. The $\overline{\Delta D_{\mathcal{G}}}$ of the *Drosophila* protein network, DMC, DMR and LPA are relatively large that cannot be ignored even when using a same root selection scheme (see Table 3). In these situations, graph curves derived from a same network have obvious differences. Fortunately, the curve that averaged over amounts of curves derived from a same network is stable with small $\overline{\Delta D_{\mathcal{G}}}$, as is shown in Figure S6b and Table 3. This result suggests that the average curve is a stable description of networks and statistically suitable for network comparison. Note that the curve difference $\overline{\Delta D_{\mathcal{G}}}$ of the first root selection scheme is very small, thus the comparisons between the PPI data and the three network models in the main text are

based on these average curves. In detail, we averaged over 100 curves derived from a same network where the root is selected by picking one end of a randomly chosen edge.

Among the four seed selection schemes, the graph curve rooted from a maximal degree vertex is the most different from the other three, which brings a variability of the graph curves, as you have predicted and figured out, that could be useful in a statistical test to compare two different graphs. We are eager to study this variability for more real-world networks and network models in the future, especially when they have a special underlying structure and are sensitive to the schemes of root selection or the graph search algorithm.

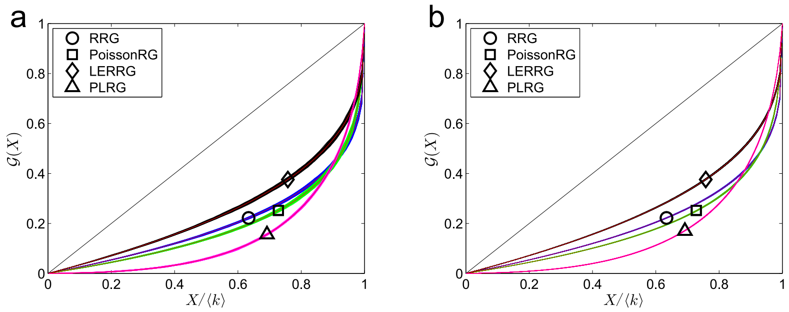

FIG. S5: **Effects of root selection for random graphs and LERRG.** Each single curve is derived from a single network by using one of the four different root selection schemes. For each one network and one scheme, we produce 100 curves, and the curves of a same network are colored same. The networks are generated by using the same parameters as in Figure 3 in the main text except the network size, in panel a, $N = 10,000$ and in panel b, $N = 100,000$.

TABLE S4: Curve difference $\overline{\Delta D_{\mathcal{G}}}$ caused by root selection schemes where network size $N = 10,000$

	RRG	PoissonRG	LERRG	PLRG
Random Edge	0.0013	0.0015	0.0018	0.0011
Random Node	0.0013	0.0014	0.0017	0.0011
Max Degree	0.0014	0.0013	0.0018	0.0010
Min Degree	0.0014	0.0015	0.0017	0.0011

Each $\overline{\Delta D_{\mathcal{G}}}$ is calculated by comparing 100 curves derived from a same network to a center curve considering different root selection schemes, where the center curve is averaged over the 100 curves using the first scheme. There are no obvious differences between the four root selection schemes. The $\overline{\Delta D_{\mathcal{G}}}$ of a LERRG is relatively larger than the random graphs since it contains a lattice structure that brings more variability during the process of BFS.

TABLE S5: Curve difference $\overline{\Delta D_{\mathcal{G}}}$ caused by root selection schemes with a larger network size, around $N = 100,000$.

	RRG	PoissonRG	LERRG	PLRG
Random Edge	0.0004	0.0005	0.0006	0.0004
Random Node	0.0004	0.0005	0.0006	0.0004
Max Degree	0.0004	0.0004	0.0006	0.0003
Min Degree	0.0004	0.0005	0.0009	0.0004

The $\overline{\Delta D_{\mathcal{G}}}$ is small and decreases with a larger network size.

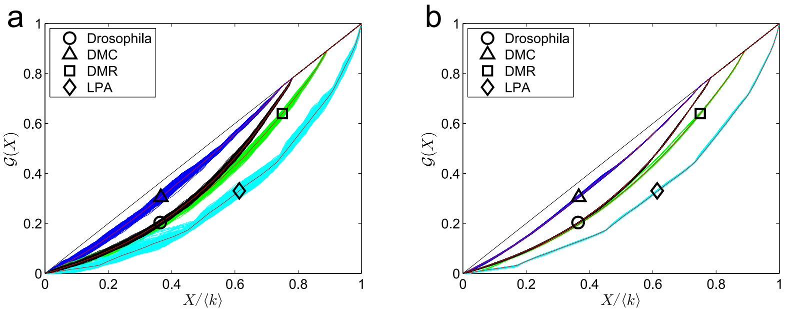

FIG. S6: **Effects of root selection for *Drosophila* PPI network and three network models.** (a) Each single curve is derived from a single network by using one of the four different root selection schemes. For one network and one scheme, we produce 100 curves, and the curves of a same network are colored same. The curves rooted from different vertices have obvious differences. The thin red curve among each curve cluster is the center curve which is averaged over the 100 curves rooted from randomly chosen edges. (b) Each single curve is an average over 100 curves derived from a same network using a same root selection scheme. For one network and one scheme, we produce 100 such average curves. The average curves are stable with smaller differences. The three networks built by models are based on the size of the *Drosophila* protein network with a confidence threshold of $P_c^* = 0.5$.

TABLE S6: Curve difference $\overline{\Delta D_{\mathcal{G}}}$ caused by root selection schemes.

	Drosophila	DMC	DMR	LPA
Random Edge	0.0029	0.0045	0.0043	0.0076
Random Node	0.0030	0.0045	0.0045	0.0076
Max Degree	0.0028	0.0053	0.0058	0.0066
Min Degree	0.0030	0.0044	0.0047	0.0077

Each $\overline{\Delta D_{\mathcal{G}}}$ is calculated by comparing 100 curves derived from a same network to a center curve considering different root selection schemes, where the center curve is averaged over the 100 curves using the first scheme. The $\overline{\Delta D_{\mathcal{G}}}$ is large that cannot be ignored.

TABLE S7: Curve difference of average curves

	Drosophila	DMC	DMR	LPA
Random Edge	0.0003	0.0004	0.0004	0.0008
Random Node	0.0004	0.0006	0.0006	0.0007
Max Degree	0.0024	0.0016	0.0058	0.0032
Min Degree	0.0005	0.0010	0.0010	0.0011

The average curves are stable with smaller differences. Graph curve rooted from the maximal degree vertex is the most different from the other three.

4. TEST THE ROBUSTNESS OF NETWORK CLASSIFICATION METHOD

To test the robustness of our classification method against noise, we artificially introduce some noise into the original network by two kinds of edge rewiring mechanisms [7, 8]. The first is to replace some percentage of original edges in the network by random ones (noise1), and the second is to randomly rewire some percentage of edges while maintaining the degree distribution of the original network (noise2).

When given a network G which is built by using one of the three network models, i.e., DMC, DMR and LPA, we perturb the structure of G by introducing different percentages of noise, and then classify the resulting networks to find back the model which built it. Figure S7 shows the median graph distances of the three network classes as functions of the rewiring percentage for the two kinds of noises, where each network instance is built by models based on the size of the *Drosophila* protein network with a confidence threshold of $P_c^* = 0.5$, and each data point is averaged over 100 different realizations of the randomization procedure. As validation, the networks are confidently classified as Poisson-distributed random graphs for high levels of the noise1. The results show that the classification performs well for small and intermediate amounts of the noises. Meanwhile, the robustness against the second noise is better than the first one since the second noise maintains the degree distribution of the original network.

Among the three network classes, the DMC networks are the most sensitive to both the two noises, even when noise2 keeps their degree distributions unchanged. The reason may be that the underlying structure of the DMC networks is sharply changed when a small fraction of noises is introduced. As shown in Figure S8, a small fraction of noises largely increases the global connectivity of the DMC networks. That is also why the classification is sensitive for the noised DMC networks. Note that a part of the noised DMC networks are classified as the DMR. The reason here could be the growth mechanisms of DMR which randomly create links between a new vertex and any other old vertices is similar in effect to noise2 which rewiring edges randomly according to the degree of vertices.

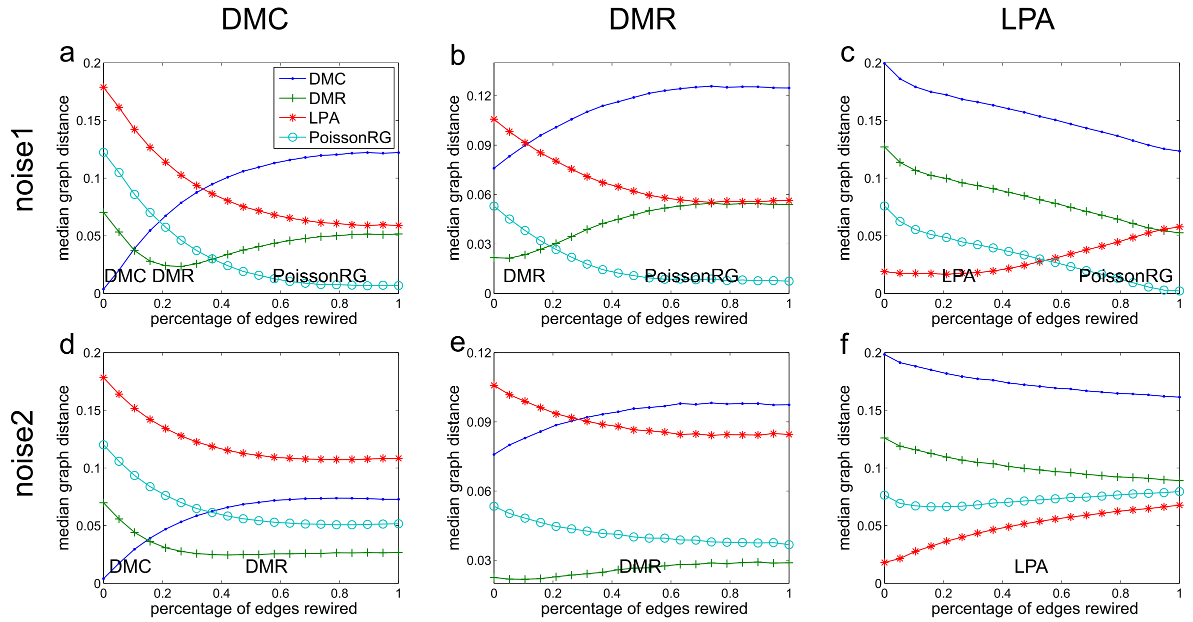

FIG. S7: **Robustness test against noises.** Classify a network G built by using one of DMC, DMR and LPA for different levels of artificially introduced noises, where the class with the minimal median graph distances wins. Noise1 is introduced by replacing original edges with random ones, and noise2 rewires edges while maintaining the degree distribution of the original network.

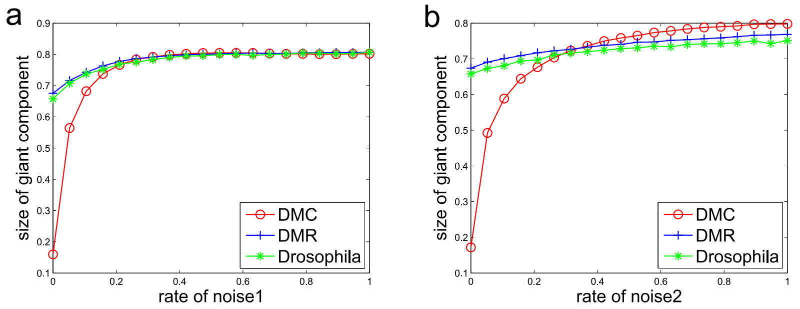

FIG. S8: The size of the giant component of the DMC networks increase rapidly when a small fraction of noises are introduced

5. NETWORK COMPARISON RESULTS

We generate 1,000 examples for each of the three different network models with their vertices number N and average degree $\langle k \rangle$ around (allowing for small intervals of $\pm 5\%$) that of the *Drosophila* network for different confidence thresholds P_c^*. The parameters q_{del}, q_{con} and q_{new} are sampled uniformly in $[0, 1]$. In each network, multiple edges and self-loops are removed and isolated vertices are eliminated. Each curve of the BFS-graph is averaged over 100 runs of BFS on the corresponding network. The results for $P_c^* = 0.5$ have been presented in the main text. The results for $P_c^* = 0.65$ and $P_c^* = 0.0$ are shown in Figures S9 and S10, respectively.

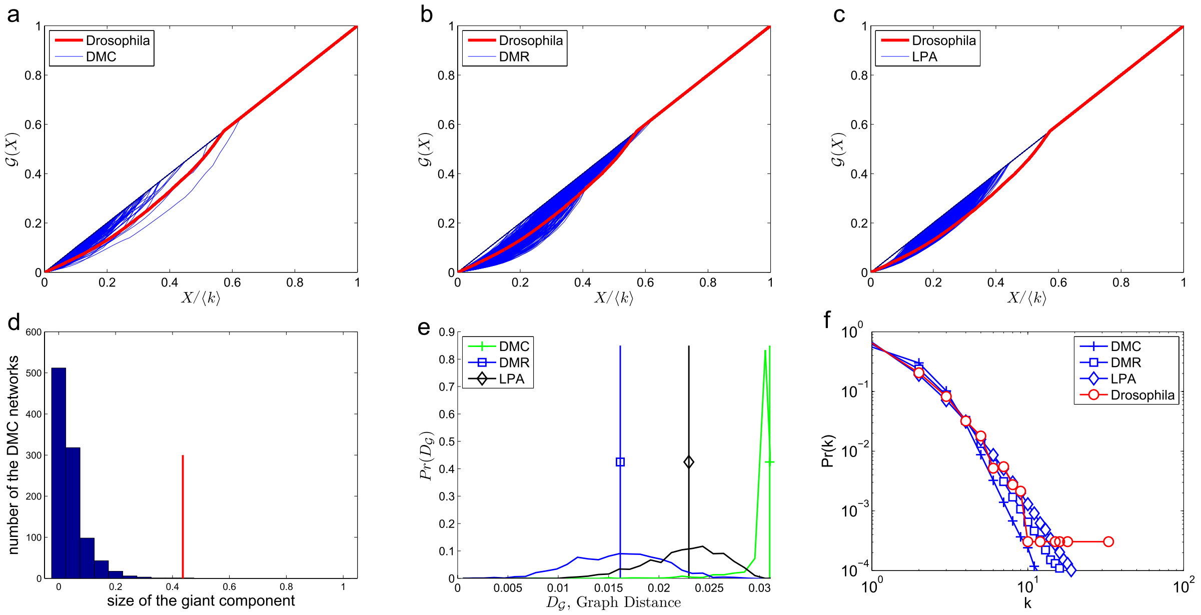

FIG. S9: Comparisons between the model networks and the *Drosophila* PPI network for $P_c^* = 0.65$. (a-c) BFS-graphs. In each diagram, the thick red curve represents *Drosophila*'s network, and the thin blue curves represent the 1,000 generated model networks. (d) The size distribution of the giant components of the 1,000 DMC networks. In *Drosophila*'s network, 44% of the vertices are in the giant component (red vertical bar). (e) Graph distance distributions. Each vertical bar represents a median graph distance $\tilde{D_{\mathcal{G}}}$ which is a value separating the closer half from the farther half to the center graph to be compared with, i.e., the *Drosophila* network. (f) Degree distributions. Each distribution of the three models is averaged over the 1,000 generated networks. Although their degree distributions are similar to that of the *Drosophila*, their curves vary widely.

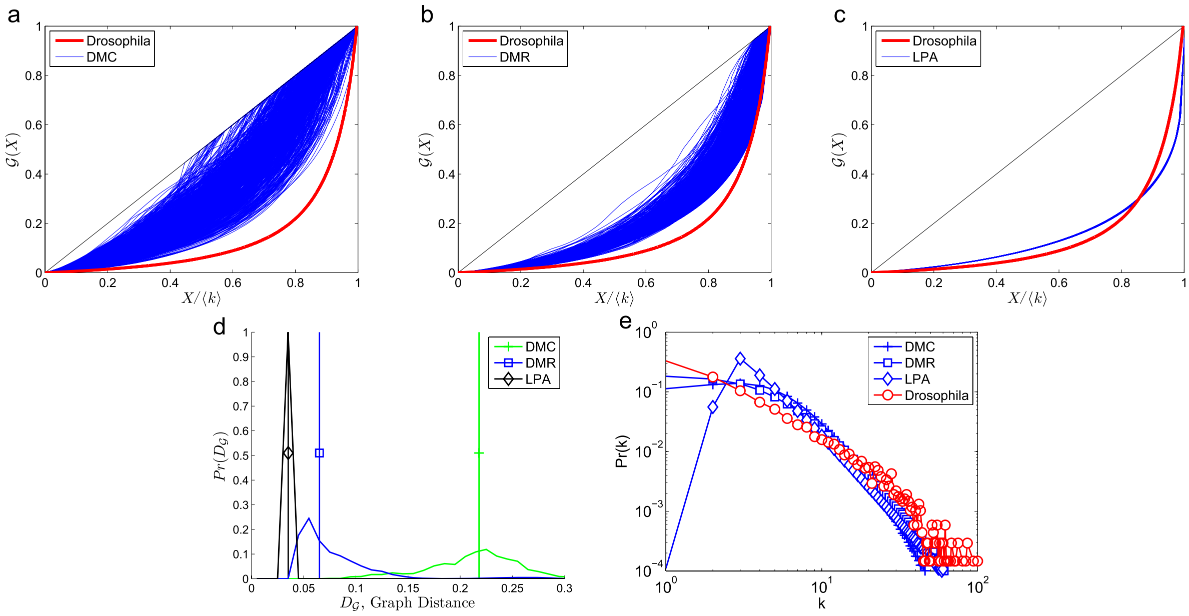

FIG. S10: Comparisons between the model networks and the *Drosophila* PPI network for $P_c^* = 0.0$. (a-c) BFS-graphs. (d) Graph distance distributions. (e) Degree distributions. Comparing with the results for higher confidence thresholds $P_c^* = 0.65/0.5$, the fits of the three models to the data for $P_c^* = 0.0$ are not good, probably due to the presence of strong additional noise in the data when including low confidence value P_c interactions.

[1] E. Isaacson and H. B. Keller, Analysis of Numerical Methods (John Wiley & Sons, New York, USA, 1966).

[2] R. L. Burden and J. D. Faires, Numerical Analysis (Brooks Cole, California, USA, 2004), 8th ed.

[3] B. Bollobás, Random Graphs (Cambridge University Press, Cambridge, UK, 2001), 2nd ed.

[4] N. C. Wormald, in Surveys in Combinatorics, 1999, edited by J. D. Lamb and D. A. Preece (Cambridge University Press, Cambridge, UK, 1999), vol. 267 of London Mathematical Society Lecture Note Series, pp. 239-298.

[5] A. Wagner, Proc. R. Soc. Lond. B Biol. Sci. **270**, 457 (2003).

[6] J. Berg, M. Lässig, and A. Wagner, BMC Evol. Biol. **4**, 51 (2004).

[7] Onnela JP, Fenn DJ, Reid S, Porter MA, Mucha PJ, et al. (2010) A taxonomy of networks. http://arxiv.org/abs/1006.5731v1.

[8] Middendorf M, Ziv E, Wiggins CH (2005) Inferring network mechanisms: The Drosophila melanogaster protein interaction network. Proc Natl Acad Sci U S A 102: 3192-3197.

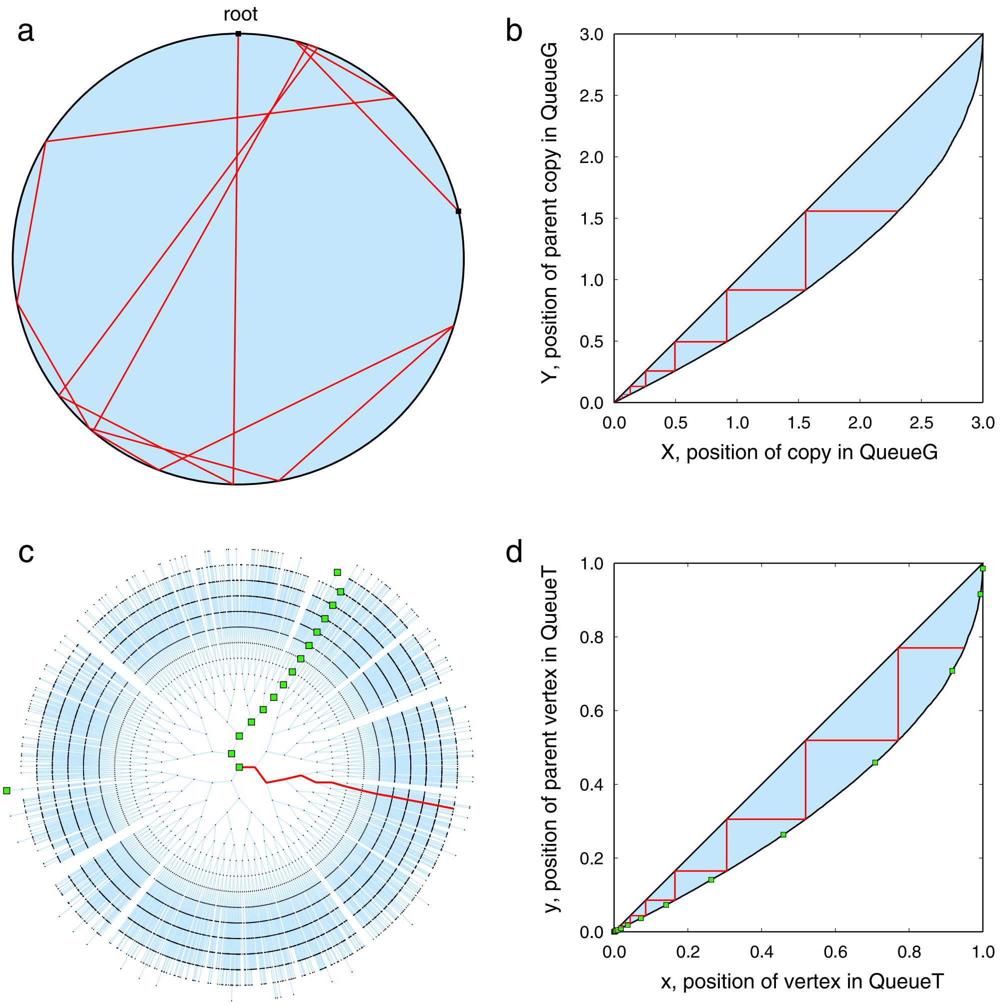

FIG. S11: The diagrams of a random r-regular graph of size $N = 10^4$ and $r = 3$. (a) Ring graph, vertices are located on a ring in random order. (b) BFS-graph, copies of vertices are located around the curve $(1 - X/3) = (1 - Y/3)^2$. (c) Radial BFS tree. (d) BFS-tree, vertices are located around the curve $(1 - x) = (1 - y)^2$. Each green square of (c) and (d) represents the last vertex in its tree level of the BFS tree. In these four diagrams, the blue parts represent the edges and the red polylines represent a same shortest path between the root and a destination node.

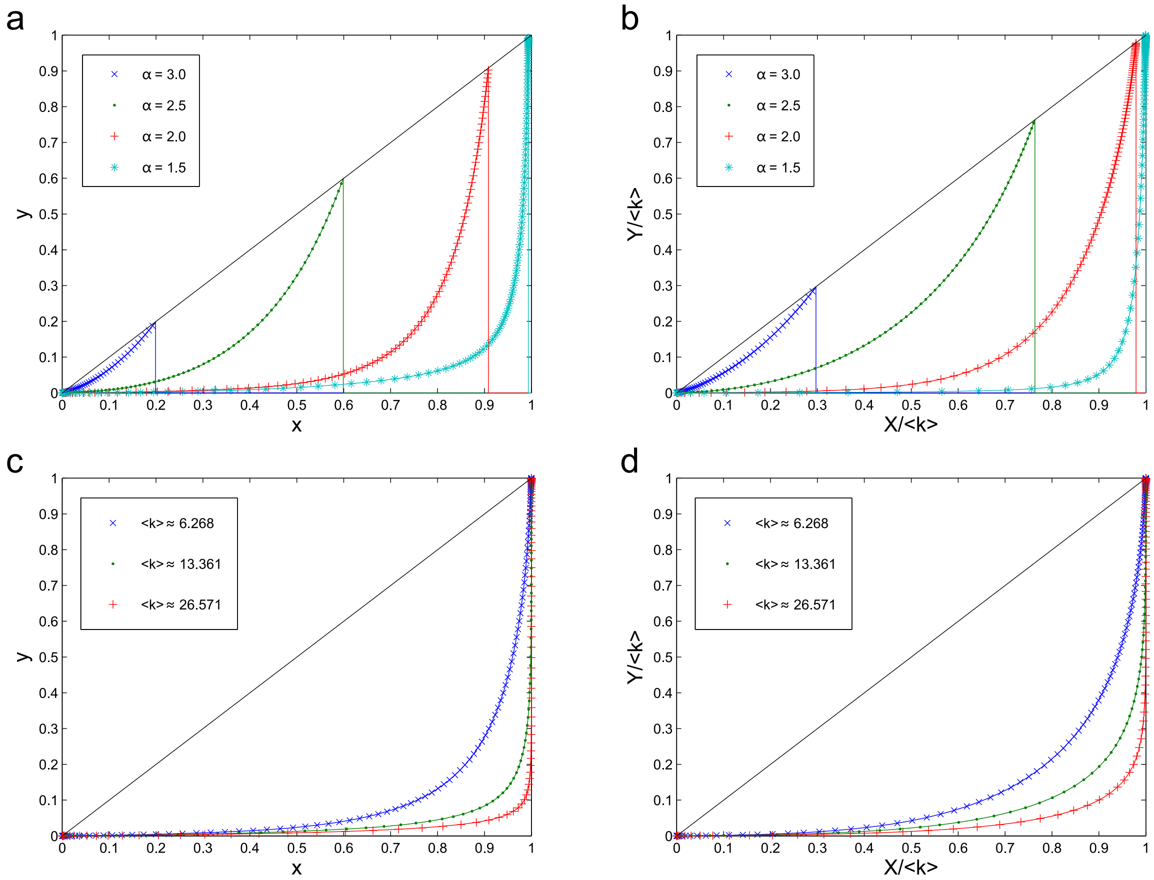

FIG. S12: BFS-trees and graphs of power-law distributed random graphs. Each solid line represents the vertices or copies resulted from one run on the associated network. The dots are obtained numerically from Eqs. S16 and S17. In (a) and (b), $N = 2,000,000$, $\alpha = 1.5, 2.0, 2.5, 3.0$; $k_{min} = 1$; $k_{max} = 1,000$. In (c) and (d), $N = 1,000,000$, $\alpha = 2.25$; $k_{max} = 1,000$; $\langle k \rangle \approx 6.268\,(k_{min} = 2)$, $\langle k \rangle \approx 13.361\,(k_{min} = 4)$, $\langle k \rangle \approx 26.571\,(k_{min} = 8)$.
